# Thermo-responsive, on-demand adhesive and tissue-conformal hydrogel electrodes for organ repair and brain-computer interfaces

**DOI:** 10.1016/j.mtbio.2025.102705

**Published:** 2025-12-20

**Authors:** Zhenchun Li, Tiantian Li, Rongfeng Ge, Feixiong Chen, Chuang Du, Dongxu Wang, Lei Wang

**Affiliations:** a Key Laboratory of Molecular Enzymology and Engineering of Ministry of Education, School of Life Sciences, Jilin University, Changchun, 130012, PR China; b Laboratory Animal Center, College of Animal Science, Jilin University, Changchun, 130062, PR China; c Disease Networks Research Unit, Faculty of Biochemistry and Molecular Medicine, University of Oulu, Oulu, 90014, Finland; d Changchun Institute of Applied Chemistry, Chinese Academy of Sciences, Changchun, 130022, PR China

**Keywords:** Hydrogel, Wet adhesion, Hemostasis, Implantable bioelectronics

## Abstract

Implantable bioelectronic devices, such as brain-computer interfaces (BCIs), face persistent challenges in achieving stable, rapid, and reversible adhesion on wet tissues due to hydration layers and mechanical mismatch, which can cause interfacial failure and unstable signals. Here, we report a conductive hydrogel interface with tissue-adaptive, temperature-controllable adhesion. The material is synthesized via dynamic co-entanglement of poly(acrylic acid) and poly(lipoic acid) with LA-NHS, establishing a dual physico-chemical anchoring mechanism that enables efficient tissue integration in aqueous environments. The hydrogel penetrates tissue microstructures within 5 s, withstands burst pressures >213 mmHg, exhibits <10 % swelling, ∼2784 % extensibility, and a low modulus of 41 kPa, thereby conforming to soft, irregular surfaces and reducing interfacial mismatch. Its temperature-triggered adhesion allows safe detachment and repositioning without apparent tissue damage, supporting repeated applications. In vivo and ex vivo tests confirm rapid hemostasis in mouse liver and tail injury models, effective sealing of porcine gastric, bladder, and intestinal defects, and stable electrocorticography and electrocardiography recordings. Moreover, the hydrogel demonstrates high cytocompatibility (>90 %), <5 % hemolysis, reactive oxygen species scavenging, and ∼90 % antibacterial efficiency. By integrating rapid wet adhesion, mechanical compliance, electrical functionality, and bioprotective features, this hydrogel provides a versatile platform for next-generation bioelectronic interfaces and soft therapeutic devices.

## Highlights

• Dual hydrophilic-hydrophobic network achieves 5 s wet adhesion with <10 % swelling.
• Cold-triggered debonding enables non-traumatic removal and repositioning.
• Conformal, low-modulus gel records stable in vivo ECoG/ECG signals.
• Ionic-liquid/Ag^+^ doping adds conductivity and ∼90 % antibacterial activity.

## Graphical abstract

**Figure:**
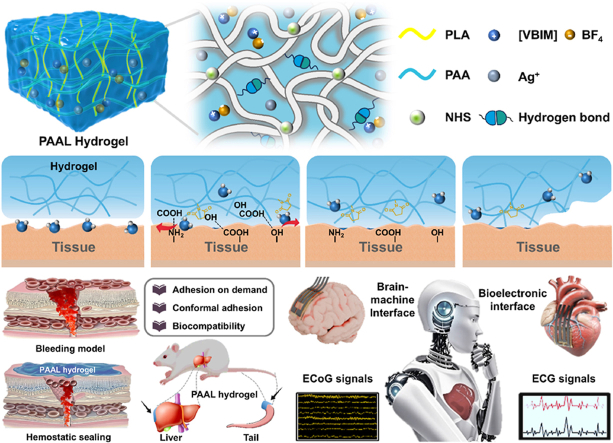

## 1 Introduction

In the field of biomedical engineering, hydrogel-based bioadhesives have garnered significant attention for applications in organ repair [[1], [2], [3]], hemostasis, human-machine interfaces [[4], [5], [6]], and implantable bioelectronics [[7], [8], [9]]. Particularly in the recording of ECoG signals and brain-computer interfaces, these materials demonstrate significant application potential due to their excellent tissue conformability and stable electrophysiological signal conduction capabilities. The unique adaptability of hydrogels to wet environments and their multifunctional integration capabilities make them one of the most promising materials for tissue repair and smart healthcare.

Their unique adaptability to wet environments and multimodal functional integration capabilities position them as promising materials for tissue repair and smart medicine [10,11]. Rapid and effective hemostasis, along with reliable tissue repair, is essential for bioadhesives [12,13]. Hydrogels are considered ideal candidates for next-generation organ repair and bioelectronic interfaces due to their outstanding interfacial adaptability, mechanical properties, and multifunctional integration [14]. Consequently, the development of novel materials with strong adhesion and compatibility in wet environments is a critical focus in current research [15]. However, achieving adequate adhesion of hydrogels on wet tissue surfaces remains a significant challenge. The interfacial hydration layer formed by blood and tissue fluids obstructs effective contact between the adhesive and the trauma surface, while water molecules often disrupt intermolecular forces, such as hydrogen bonds and electrostatic interactions, leading to adhesion failure [[16], [17], [18]]. This presents difficulties in meeting the demand for immediate sealing in high-pressure bleeding scenarios. To address the adhesion problem in wet environments, the wet adhesion behavior across various liquid environments has been enhanced by modifying the hydrophilic and hydrophobic polymer chains on the hydrogel surface [[19], [20], [21]]. For instance, Wang et al. developed an asymmetric Janus hydrogel bioadhesive that incorporates acrylic acid (AA) as a hydrophilic monomer, characterized by its abundant free carboxyl groups. In contrast, lauryl methacrylate (LMA), which features long carbon chains, and hexadecyl trimethyl ammonium bromide (CTAB), a surfactant, were employed as hydrophobic monomers and surfactants, respectively. Furthermore, [2-(methacryloyloxy)ethyl]dimethyl-(3-sulfopropyl)ammonium hydroxide (DMAPS) was introduced as a copolymerized monomer to enhance the adhesion stability of the hydrogels in wet environments by facilitating electrostatic interactions within the hydrogel networks [22].

The swelling resistance of hydrogels is a fundamental property for their applications in wet physiological environments, as it directly influences the stability and durability of the adhesion interface [23,24]. Excessive swelling reduces the modulus of the hydrogel and increases the mechanical mismatch with the surrounding tissue, ultimately leading to debonding. In this context, Shen et al. leveraged the unique coupling structure of carboxyl and phenyl groups in N-acryloyl phenylalanine (APA), an amino acid derivative, to facilitate interfacial drainage and enhance matrix toughness. They also harnessed various electrostatic interactions mediated by zwitterionic polymers to develop a novel hydrogel adhesive. This adhesive demonstrates excellent tissue adhesion and matrix swelling resistance under humid conditions, exhibiting a swelling rate of only 4 % [25]. Furthermore, it is noteworthy that on-demand desorption properties are crucial for hydrogel adhesives, as a strong adhesive may inadvertently cause secondary damage to the tissue during the removal process. Wu et al. proposed a temperature-mediated controllable adhesive P(AAm-co-NIPAM-co-OMA)/PTA-Fe hydrogel (PANC/T-Fe) that is based on dynamic interchain interactions and exhibits enhanced wet adhesion properties. This hydrogel can be removed on demand simply by applying ice. At room temperature, the PANC/T-Fe hydrogel becomes soft and exhibits strong adhesion; once applied to regular glass, it remains firmly attached and is difficult to remove. When cooled to a lower temperature (5 °C) with an ice pack, the gel's cohesion increases and it becomes more elastic, allowing for easy peeling. Hydrogels have emerged as ideal materials for implantable bioelectronic interfaces due to their excellent flexibility, biocompatibility, and wet adhesion [[26], [27], [28]]. Recent developments in soft and self-healing bioelectronic systems further underscore the importance of mechanically compliant interfaces for stable neural signal recording [[29], [30], [31]]. Achieving mechanical modulus matching with soft tissues and conformal contact allows for seamless integration with the irregular surfaces of biological tissues and adaptation to dynamic deformations, thereby enabling efficient and stable signal transmission and energy exchange in implantable bioelectronic interfaces [[32], [33], [34]]. Wang et al. designed a bioadhesive ultra-soft brain-machine interface (BMI), which integrates a bioelectronic device with dopamine methacrylate-hybridized poly(3,4-ethylenedioxythiophene) nanoparticles (dPEDOT NPs) within a highly conductive hydrogel. This hydrogel possesses strong adhesive properties that enable it to bind tightly to metallic microcircuits and seamlessly adhere to brain tissue, facilitating high-quality signal recording and extending the lifespan of BMIs in clinical settings [15]. Injectable conductive hydrogels have likewise emerged as minimally invasive tissue-interfacing materials that can form conformal and conductive networks in situ, enabling stable electrophysiological coupling with deep or irregular tissues [35,36].

In this paper, we present a wet-adhesive, swelling-resistant hydrogel designed for on-demand adhesion, which is prepared using chain segment entanglement and dynamic diffusion strategies. By forming an interpenetrating network structure between PLA, poly(lipoic acid) N-hydroxysuccinimide (PLA-NHS), and PAA, the resultant PAAL hydrogels were prepared and exhibited good wet adhesion and swelling resistance. These components entangle with one another. The hydrophilic groups of PAA synergize with the hydrophobic PLA, enabling dynamic adaptation to complex, rough, or curved surfaces through pressure-induced chain segment rearrangement. This results in mild and instant adhesion on wet surfaces, increases the contact area with the substrate, and facilitates physical attachment through non-covalent interactions within the network, such as hydrogen bonds and electrostatic interactions. Furthermore, the synergistic covalent bonds between NHS on the adhesion side and amino groups on the organ tissue side allow the hydrogel to establish strong adhesion to the tissue. The hydrophobic lipoic acid chain segments of PLA inhibit the infiltration of water molecules into the network due to their hydrophobic nature, thereby repelling water at the interface and preventing excessive water absorption, which could lead to the detachment of the adhesion layer. Additionally, the dynamic covalent bonds of PLA (disulfide bonds) and physical interactions (hydrogen bonds, π-π stacking) effectively safeguard the dynamic bonding network from excessive swelling due to water intrusion, thus preserving its ability to reorganize and enhancing interfacial adhesion. Moreover, the temperature-mediated adhesive strength is tunable, ensuring that the hydrogel achieves effective wet adhesion in liquid environments while allowing easy de-adhesion via ice compression, which helps minimize secondary damage to the organ tissue injury. Incorporating ionic liquids into PAAL hydrogels imparts high electrical conductivity, while the addition of AgNPs further enhances conductivity and provides excellent antimicrobial properties. The compatibility of the conductive hydrogel with metallic microcircuits enables seamless adhesion to brain tissue by forming intimate conformal contact. Notably, integrated conformal hydrogel for biointerfacial electrodes (CHBE) is utilized for ECoG signal recording and implantable ECG signal monitoring, providing improved stability and minimizing tissue damage compared to conventional rigid electrodes. In summary, PAAL hydrogels, which are prepared through a simple and cost-effective process, exhibit stable wet adhesion properties, making them a promising candidate for future applications in organ repair and implantable bioelectronics.

## 2 Results and discussion

### 2.1 Design and characterization of PAAL hydrogels

**Fig. 1:**
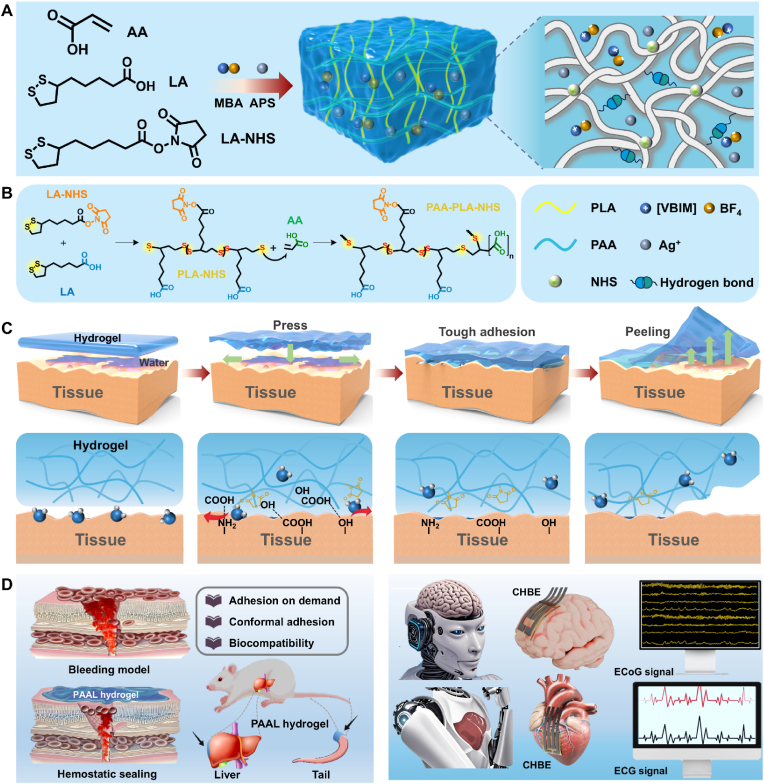
A) Schematic diagram of the preparation of PAAL hydrogels. B) Diagram of the crosslinking mechanism in PAAL hydrogels. C) Schematic diagram of conformal adhesion between PAAL hydrogel and wet tissue at the interface. D) Schematic diagrams showing the application of PAAL hydrogel for hepatic and caudal hemostasis in a murine model, as well as for electrocorticography (ECoG) signal recording and implanted electrocardiography (ECG) signal monitoring.

Conformal contact hydrogel bioelectrode interfaces are designed to enhance swelling resistance and improve adhesion in wet environments [37]. In this study, we propose a strategy involving chain segment entanglement and dynamic diffusion, which synergistically introduces hydrophobic and hydrophilic components to improve interfacial adhesion stability by promoting interfacial interactions (Fig. 1A–D). Acrylic acid (AA) was incorporated as a hydrophilic monomer, while lipoic acid (LA) and LA-NHS were selected as hydrophobic monomers. Notably, the presence of NHS keeps the poly(lipoic acid) chain stable and inhibits its depolymerization. In this regard, the copolymerization of LA with LA-NHS was named PLA to make it easier to understand. Additionally, the ionic liquid 1-vinyl-3-butylimidazolium tetrafluoroborate (VBIMBF_4_) was introduced to establish an ionic conduction pathway, because its vinyl structure enables good integration into the polymer network and its ion pair provides stable and efficient ion transport. Its compatibility with both hydrophilic and hydrophobic domains further supports continuous ionic conduction within the hydrogel. Meanwhile, the incorporation of AgNPs enhances the conductivity of the hydrogel and contributes to its antimicrobial performance. These components were sequentially added to create a homogeneous solution, followed by the addition of the crosslinking agent N,N′-methylenebisacrylamide (MBA) and the initiator ammonium persulfate (APS), with continued mixing to yield a hydrogel precursor solution. This precursor solution was then poured into a mold and crosslinked at 70 °C for 4 h. The entire process was conducted in an open environment and included heating. The PAAL hydrogel was successfully obtained. The heating and dehydration during preparation facilitated the aggregation of active ester groups (-NHS) and carboxylic acid groups at the interface, leading to the formation of covalent bonds with amino, sulfhydryl, and other functional groups on the skin surface, establishing robust chemical linkages. Furthermore, the hydrophobic segments of the LA chains aggregated under thermodynamic driving forces, forming hydrophobic microdomains that anchor to the substrate via hydrophobic interactions.

**Fig. 2:**
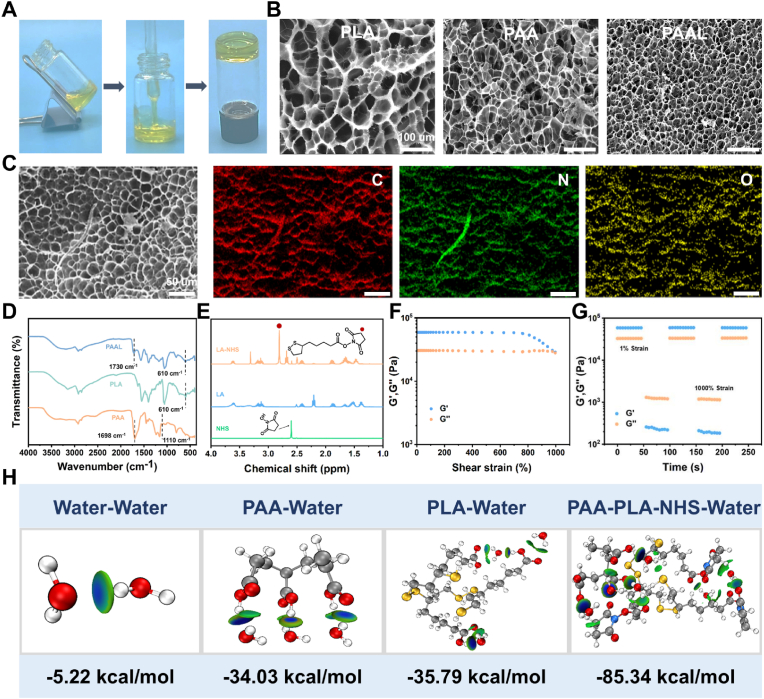
Characterization and analysis of the PAAL hydrogel. A) Schematic diagram of the polymerization process in a vial. B) SEM images and pore size distribution of PLA, PAA and PAAL hydrogels. C) Elemental mapping images of the PAAL hydrogel. D) FT-IR spectra of PAA, PLA and PAAL hydrogels. E) ^1^H NMR spectra of LA, NHS and LA-NHS. F) Modulus-strain curve of the PAAL hydrogel. G) G′ and G″ of the PAAL hydrogel during three cycles between 1 % and 1000 % strain. H) The binding energy of each component to water was calculated by DFT.

The hydrogel enables on-demand adhesion and controlled peeling. In wet physiological conditions, the strength of interfacial adhesion can be flexibly regulated by temperature changes, such as ice application, which reduces adhesion at low temperatures and helps prevent secondary damage to the wound during removal. The hydrophobic chain segments minimize the interference of the hydration layer by displacing interfacial water molecules, while the carboxylic acid groups of PAA form multiple hydrogen bonds and electrostatic interactions with the polar groups (-OH, -NH_2_) on the tissue surface, facilitating a rapid contact adhesion process. Simultaneously, this interaction prevents water molecules from infiltrating the network via the hydrophobic chains, thereby enhancing interfacial adhesion. Furthermore, the addition of ionic liquid creates a conductive pathway, allowing the hydrogel, with its high conductivity, to function as a conformal electrode that can seamlessly adhere to the brain cortex or myocardium for stable recording of ECoG or ECG signals. Notably, this system constructs a hydrophobic-hydrophilic interpenetrating network directly through a green aqueous copolymerization process, reducing the risk of toxicity associated with organic solvents used in traditional hydrophobic modifications, while maintaining biocompatibility and environmental sustainability. The hydrogel exhibits multifunctionality through the synergistic effects of dynamic bonding, exclusion of interfacial water layers, and hydrophobic repulsion. As shown in Fig. 2A, the precursor solution rapidly undergoes polymerization in the vial, forming a hydrogel. Scanning electron microscopy (SEM) demonstrates that the PAAL hydrogel possesses a uniform and well-defined porous structure, with a denser pore size compared to PAA and PLA hydrogels. This confirms the formation of more chain-knot entanglements via non-covalent interactions, resulting in enhanced structural density (Fig. 2B). Energy-dispersive spectroscopy (EDS) mapping (Fig. 2C) further reveals distinct distributions of carbon (C), nitrogen (N), and oxygen (O) elements. The FT-IR spectrum of the LA monomer shows a characteristic C=O stretching vibration peak at 1693 cm^−1^ and a prominent S-S bond peak at 519 cm^−1^. Notably, in the PLA hydrogel, the S-S peak at 519 cm^−1^ disappears, while the C-S bond peak broadens and shifts to 610 cm^−1^, indicating successful polymerization and formation of extended polymer chains. In addition, the PAA hydrogel displays C-H and C-O stretching peaks at 1698 cm^−1^ and 1110 cm^−1^, respectively. However, the 1110 cm^−1^ peak is absent in the PAAL hydrogel, possibly due to the formation of a new hydrogen bonding network between PAA and PLA chains (Fig. 2D; Fig. S1, Supporting Information).

The results of ^1^H NMR analysis confirmed the successful incorporation of NHS-activated esters, as evidenced by the characteristic peak at 2.80 ppm (Fig. 2E). Furthermore, the presence of hydrophobic chains, in combination with the NHS ester components, contributes to the increased surface hydrophobicity of the PAAL hydrogel, which exhibits a contact angle of 96°. This enhanced hydrophobicity facilitates the repulsion of interfacial water layers (Fig. S2, Supporting Information), thereby enhancing interfacial adhesion. In this work, we demonstrate that NHS-activated esters can effectively stabilize PLA against depolymerization. The resulting PAAL hydrogels remained homogeneous and transparent even after two days of storage at room temperature, with no observable crystallization peaks in the X-ray diffraction (XRD) patterns (Figs. S3 and S4, Supporting Information).

Rheological tests further investigated the viscoelastic and self-healing properties of PAAL hydrogels. Analyses conducted through frequency scanning tests indicated that the storage modulus (G′) consistently surpassed the loss modulus (G″) across over the entire time-scan range (0–100 s), underscoring its predominantly elastic response (Fig. S5, Supporting Information). Strain amplitude scanning tests were employed to explore the linear viscoelastic region within the 1–1000 % strain range. The intersection of the G′ and G″ curves occurred at a strain of 974 %, indicating that the loss modulus (G″) gradually exceeded the energy storage modulus (G′), leading to the disruption of the hydrogel's network structure. To assess the self-healing capability of the PAAL hydrogel, a continuous step strain test was performed. When the strain reaches 1000 %, the shear-thinning behavior of the PAAL hydrogel shows G″ > G′. When the strain returns to 1 %, both G′ and G″ promptly revert to their original values (Fig. 2F and G). This demonstrates that PAAL hydrogels possess excellent self-healing ability, which facilitates the adhesion process at the interface. We also conducted density-functional theory (DFT) calculations (Fig. 2H). The DFT analysis revealed the interaction energies of various substances with water, with PAA-PLA-NHS exhibiting the highest binding energy with water, reaching −85.34 kcal/mol. This suggests that strong interactions with water molecules can occur, and the synergistic effect between them significantly impacts the surface of wet mucous skin. Such highly intensive interactions can greatly disrupt the hydrogen bonding network of water, thereby contributing to adhesion at the wet environmental interface.

### 2.2 Adhesion properties of PAAL hydrogels

**Fig. 3:**
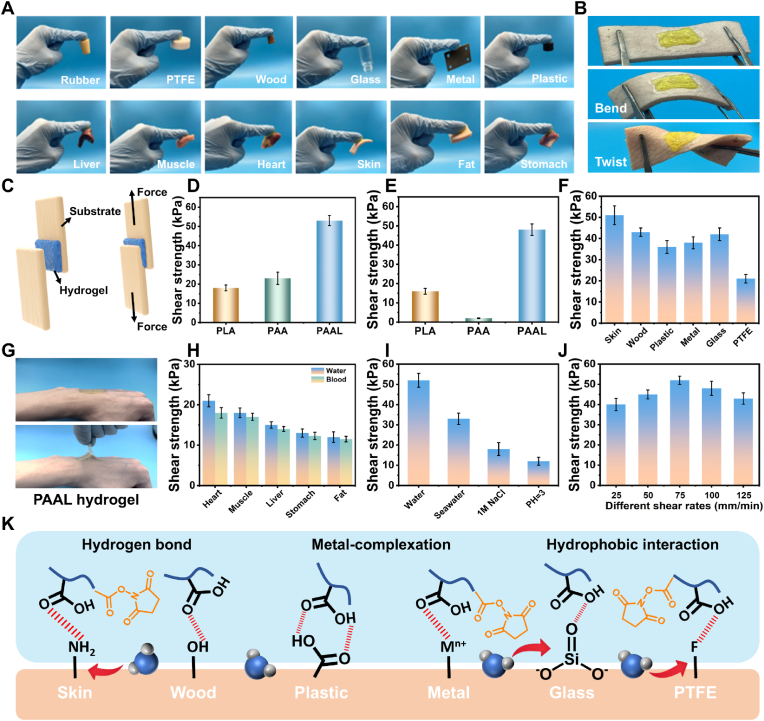
Adhesion performances of the PAAL hydrogel. A) Photographs of PAAL hydrogel adhesion to different materials in air. B) Different states of PAAL hydrogel adhesion on pigskin surface. C) Schematic diagram of adhesion shear strength test of hydrogel. D) Shear adhesion strength of PLA, PAA and PAAL hydrogels in air. E) Shear adhesion strength of PLA, PAA and PAAL hydrogels underwater. F) Shear adhesion strength of PAAL hydrogels on different substrates. G) Pictures of strong adhesion to human skin. H) Shear adhesion strength to different isolated tissues in aqueous and blood-moistened environments. I) Shear adhesion strength in different aqueous solutions. J) Shear adhesion strength at different tensile speeds. K) Formation of possible non-covalent interactions between PAAL hydrogels and different solid substrates.

Superior adhesion is a critical metric for hydrogel adhesives [38,39]. Initially, we evaluated the adhesion performance of the PAAL hydrogel. As illustrated in Fig. 3A, it demonstrated strong adhesion to various solid substrates, including rubber, PTFE, wood, glass, metal, and plastic, achieving robust adhesion in air. Similarly, effective adhesion to biological tissues was observed, with successful adhesion to liver, muscle, heart, skin, fat, and stomach tissue. Fig. 3B visually confirms that the PAAL hydrogel maintained strong adhesion to porcine skin without any separation, even when subjected to bending and twisting. Furthermore, as depicted in Fig. 3C, we assessed the adhesion performance of PAAL hydrogels using a lap-shear test. The adhesion strengths of different hydrogel samples were quantitatively evaluated, revealing that in air, the shear adhesion strengths of PLA, PAA, and PAAL were 18 ± 0.74 kPa, 23 ± 1.33 kPa, and 54 ± 1.21 kPa, respectively (Fig. 3D). We also tested the shear adhesion strengths in water, finding that the adhesion strengths of PLA and PAA hydrogels exhibited poor wet adhesion performance, particularly for PAA, which measured only 3 kPa. Notably, the underwater adhesion strength of PAAL remained nearly unchanged, demonstrating excellent underwater wet lap strength (Fig. 3E).

The bond strength of adhesives is affected by cohesion and adhesion forces. Adhesive failures of hydrogel adhesives are attributed to failure of the adhesion interface and internal fracture of the adhesive due to poor mechanical properties. The optimal bond strength is achieved when balancing the cohesive forces inside the hydrogel adhesive with the adhesive forces on its surface [40]. We selected hydrogels with different ratios (AA:LA/LA-NHS) for mechanical property testing, and the results showed that the maximum tensile strength of the optimal ratio was 87 kPa and the elongation was 2784 %. Notably, the Young's modulus was 41 kPa, which is closer to the modulus of human tissues and is conducive to stronger adhesion to tissues and comfort (Figs. S6 and S7, Supporting Information). In addition, we further investigated the influence of different heating retention times on the hydrogel properties by inducing hydrotropic evaporation. As the heating time increased from 1 h to 6 h, the hydrogel progressively exhibited higher modulus and reduced stretchability (Figs. S8 and S9, Supporting Information). When the heating time reached 6 h, partial dehydration caused the hydrogel network to harden and lose flexibility, which impaired its conformal contact with irregular tissue surfaces and consequently reduced its adhesion capability. We investigated the underwater adhesion properties of various substrates, as illustrated in Fig. 3F. The PAAL hydrogel exhibited strong underwater adhesion to a range of materials, including wood, plastic, skin, metal, glass, and PTFE. Notably, the highest underwater shear adhesion strength of 51 kPa was recorded for skin. As demonstrated in Fig. 3G, when PAAL hydrogel was adhered to the skin surface, it rapidly formed a robust bond and could be easily lifted while adhering to the human skin. To further evaluate the adhesive performance under clinically relevant bleeding conditions, shear adhesion tests were conducted on ex vivo tissues covered with either water or blood. As shown in Fig. 3H, the adhesion strength on blood-covered porcine skin (21.5 kPa) was only slightly different from that on water-moistened porcine skin (18.7 kPa), and was at a similar level to that observed on other water-rich organ tissues. This demonstrates the strong tolerance of the material's adhesive performance in blood environments, confirming its effectiveness even under blood-rich conditions. This can be attributed to the rapid displacement of blood by the hydrophobic segments and the simultaneous anchoring achieved through multiple physical and dynamic covalent interactions with the underlying tissue. Additionally, PAAL hydrogels were subjected to harsh aqueous solvent environments, including seawater, 1 M NaCl, and aqueous solutions at pH 3 (Fig. 3I). The reduced adhesion strength of PAAL hydrogels in aqueous solutions at pH 3 can be attributed to the acidic conditions, which disrupt the hydrogen bonds and hydrophobic interactions within the polymer networks.

To investigate the effect of shear rate on underwater adhesion strength, we conducted shear adhesion rate tests at various tensile rates (25–125 mm/min). The adhesion force increased progressively with the tensile rate, achieving a maximum value of 53.4 ± 1.34 kPa at 75 mm/min. This finding indicates that the PAAL hydrogel can withstand destructive stresses at varying rates when subjected to externally induced tensile forces (Fig. 3J). The remarkable generalized adhesion can be attributed to the multiple physical interactions involved in the adhesion mechanisms of PAA and PLA-NHS chains. In this regard, we further explored the adhesion strength at different ratios, and the results showed that the shear adhesion strength was the highest when the ratio was 3:3, and the adhesion strength of the hydrogel showed an increase and then a decrease as the heating induced the water evaporation (Figs. S10 and S11, Supporting Information). As the heating time increases, partial dehydration compacts the internal network of the hydrogel, as shown in Figs. S12–13 (Supporting Information). This densification reduces the hydrogel's compliance and limits its ability to conform to irregular surfaces, leading to decreased adhesion strength at longer heating durations. Specifically, the strong hydrophilicity of PAA facilitates a connection with the wetting interface through its carboxyl groups, which reduces interfacial energy and enhances wettability. Meanwhile, the hydrophobic ester groups of PLA-NHS encourage the hydrogel to expel interfacial water molecules upon contact with the wetted surface, thereby minimizing the hydration layer's interference. This process enables the repulsion of surface water molecules and protects the adhesive interface, resulting in a durable underwater wet adhesion. As illustrated in Fig. 3K, when the PAAL hydrogel contacts the wet substrate, the hydrophilic carboxyl groups form a hydrogen bonding network with the substrate, while the hydrophobic ester groups repel interfacial water molecules, establishing hydrophobic interactions. This combination generates multiple non-covalent interactions between the hydrogel and the substrate, leading to stable and robust interfacial adhesion.

### 2.3 Burst pressure and tissue adhesion of PAAL hydrogels

**Fig. 4:**
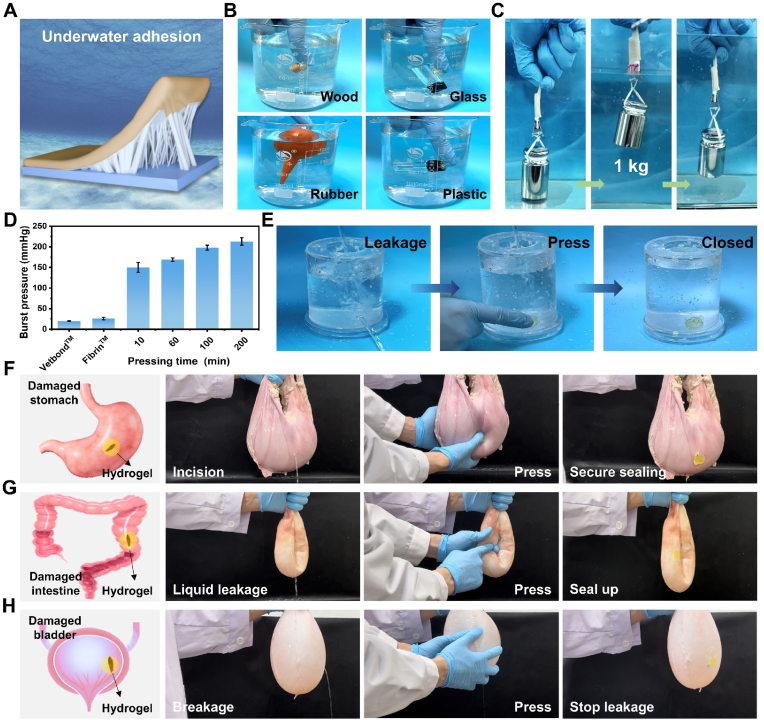
Underwater adhesion properties of the PAAL hydrogel. A) Schematic representation of the PAAL hydrogel adhesion to solid substrates underwater. B) Photographs of the PAAL hydrogel adhesion to different materials underwater. C) Photographs of the PAAL hydrogel bonded pigskin lifting a 1 kg weight underwater. D) Comparison of burst pressures of commercially available adhesives and PAAL hydrogel at different bonding times. E) PAAL hydrogel is used to plug leaky gaps under pressure. F)Pressure sealing of porcine stomach wounds with the PAAL hydrogel. G) Sealing of pig intestines without leakage. H) In vitro sealing of porcine bladder leakage holes with the PAAL hydrogel.

Achieving robust adhesion of hydrogels to wetted tissue surfaces is crucial for their application as tissue adhesives [41,42]. As illustrated in Fig. 4A, hydrogels exhibit significant adhesive strength at the bonding interface when submerged. Initially, we evaluated the adhesion performance on various materials in an underwater setting. Fig. 4B indicates that materials such as wood, glass, rubber, and plastic can be easily adhered to in an aquatic environment. Furthermore, Fig. S14 (Supporting Information) visually confirms that the PAAL hydrogel maintains strong adhesion to wet pig skin, with no separation occurring even when subjected to a vigorous stream of water. The long-lasting underwater adhesion is due to the excellent underwater swelling resistance of PAAL hydrogel (<10 %), which resists water damage to the interior of the network, thus maintaining long-term adhesion (Fig. S15, Supporting Information). Notably, the two pieces of pig skin adhered to the PAAL hydrogel lap were able to support a weight of 1 kg without failure when pressed by a 500 g weight for less than 20 s, thereby demonstrating the hydrogel's excellent adhesion properties in aqueous conditions (Fig. 4C).

We evaluated PAAL hydrogel against two commercially available bioadhesives, Vetbond and Fibrin, in underwater burst pressure tests. The results, presented in Fig. 4D, indicate that the commercially available bioadhesives exhibited low adhesion performance on wet pigskins, which hindered their ability to maintain strong adhesion under high burst pressures. In contrast, PAAL hydrogel demonstrated exceptional burst pressure resistance, as the pressurization time increased from 10 min to 200 min, it withstood a burst pressure of 213 ± 9.23 mmHg. This robust burst pressure resistance is crucial for effectively sealing tissues damaged by burst pressure in vivo. Notably, the performance of the PAAL hydrogel after 10 min of compression was 7.53 and 5.76 times greater than that of Vetbond and Fibrin, respectively. Furthermore, PAAL hydrogel exhibited superior adhesion to wet skin tissue compared to previously reported hydrogel bioadhesives (see Table S1, Supporting Information). These findings suggest that the PAAL hydrogel holds significant promise as a bioadhesive for applications requiring hemostasis under high burst pressure. In this context, the PAAL hydrogel was applied to a leaky port of a cup that was continuously filled with water to assess its pressurized adhesion capabilities. Subsequent observations confirmed that the cup did not exhibit any further leakage following the adhesion repair, thereby demonstrating the hydrogel's rapid and strong adhesion properties to wetted surfaces. This provides a solid foundation for the development of high-performance sealing materials (Fig. 4E; Movie S1, Supporting Information). Additionally, we affixed the PAAL hydrogel to a commercial electrode on the arm, while the commercial electrode was easily detached under the impact of water flow, the PAAL hydrogel remained securely adhered, showcasing its resilience under dynamic conditions (Fig. S16, Supporting Information).

It is noteworthy that the underwater bond strength of the PAAL hydrogel increased to 60.7 kPa when the temperature was raised to 45 °C. This enhancement is attributed to the dehydration and contraction of the hydrogel network, which increases the contact pressure between the polymer chains and the substrate, thereby enhancing mechanical interactions. Simultaneously, this contraction generates a preloading force that inhibits interfacial peeling. Conversely, when the bonding temperature was decreased to 5 °C, the underwater bond strength diminished to 13 kPa, resulting in a lower interfacial bond strength (Fig. S17, Supporting Information). This reduction is due to the restricted movement of molecular chains within the network at lower temperatures, which hampers the network's ability to effectively adapt to interfacial deformation. Additionally, the hydrophobic effect is weakened, complicating the repulsion of interfacial water molecules, leading to the formation of a hydration layer between the adhesive and the substrate. This layer reduces the effective contact area and contributes to adhesion failure. Based on this temperature dependence, PAAL hydrogels demonstrate on-demand detackification (Fig. S18, Supporting Information). The mechanism of on-demand debonding relies on the temperature-induced reversible activation and freezing of the dynamic bonding network, which includes hydrogen bonding, hydrophobic interactions, and disulfide bonding. At elevated temperatures, PAAL hydrogels achieve strong adhesion through enhanced interfacial bonding and stress dissipation, whereas controlled debonding is initiated by the inactivation of the dynamic bonding and the formation of an interfacial hydration layer at lower temperatures (Fig. S19, Supporting Information).

In addition, to evaluate PAAL hydrogel's effectiveness for sealing wet and dynamically responsive biological tissues, we employed porcine-based ex vivo organ models comprising the stomach, intestine, and bladder, providing physiologically relevant surfaces that mimic the conditions of in vivo organ damage. As shown in Fig. 4F, PAAL hydrogel successfully adhered to a water-filled perforated bladder (Diameter: 5 mm) for 5 s without leakage when pressed, confirming its capability to form a wound seal on the perforated bladder (Movie S2, Supporting Information). We created a 5 mm incision in the water-filled bowel, and the PAAL hydrogel provided a rapid, firm seal on the perforated bowel with no fluid leakage observed under compression (Fig. 4G; Movie S3, Supporting Information). Furthermore, PAAL hydrogel was able to maintain a press seal on a water-filled perforated bladder (5 mm in diameter) with continuous water input (Fig. 4H).

### 2.4 Hemostatic properties and conformal adhesion interface

**Fig. 5:**
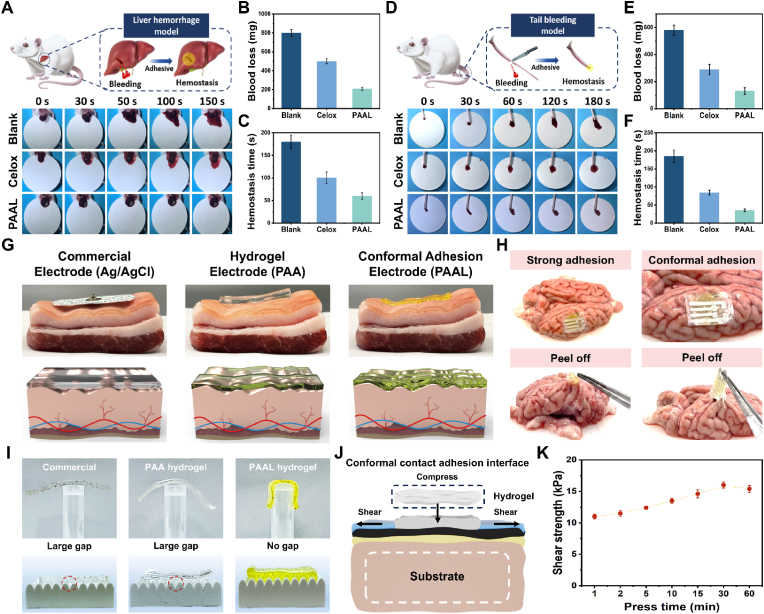
A) Diagram of the process of hemostasis of liver loss in mice by PAAL hydrogel, and photographs of the hemostatic process in the blank group and chitosan hemostatic powder group. B) Comparison of PAAL hydrogel, blank group and chitosan hemostatic powder on blood loss in liver injury. C) Comparison of hemostasis time. D) Diagram of the hemostatic process of mouse tail amputation, and photographs of the hemostatic process in the blank group and chitosan hemostatic powder group. E) Comparison of blood loss in tail injury. F) Comparison of hemostasis time. G) Adhesion of commercial electrodes, PAA hydrogel and PAAL hydrogel to porcine skin surface. H) Adhesion and conformal contact of PAAL hydrogels with printed electrodes on the brain surface. I) Visualization of fit on rough surfaces. J) Schematic representation of conformal adhesion and adhesion to wetted rough surface. K) Shear adhesion strength of PAAL hydrogel to moist brain tissue at different pressing times.

The liver is particularly vulnerable to trauma, leading to significant bleeding. We evaluated the hemostatic effect of the PAAL hydrogel in an in vivo rat liver injury model, where the injury was approximately 5 mm in diameter. For comparison, we applied a commercial chitosan hemostatic powder to the bleeding wound as a control. As illustrated in Fig. 5A, the filter paper placed beneath the liver in the PAAL hydrogel group exhibited a relatively small bloodstain area after 150 s of continuous observation. The results regarding blood loss and hemostasis time are presented in Fig. 5B and C. In the blank liver defect group, the average blood loss was 802 mg, with a hemostasis time of 180 s. In contrast, the group treated with commercial chitosan hemostatic powder demonstrated reduced blood loss and shorter hemostasis time. Notably, the PAAL hydrogel group achieved more significant hemostasis, with only 210 mg of blood loss and a hemostasis time of 57 s. These findings indicate that the PAAL hydrogel has a pronounced effect on rapid hemostasis in the liver. Additionally, we evaluated the hemostatic effect of the PAAL hydrogel using a rat tail defect model in vitro. The results of the test are presented in Fig. 5D, which indicates that a continuous observation period of 180 s was conducted. Notably, the filter paper positioned beneath the tail in the PAAL hydrogel group exhibited a smaller bloodstain area. This finding aligns with the injury results. Specifically, detailed data can be found in Fig. 5E and F. The PAAL hydrogel group demonstrated a markedly enhanced hemostatic effect, with a blood loss of merely 126 mg and a hemostasis time of 36 s. In comparison to the blank group, the hemostasis time and blood loss in the PAAL hydrogel group were significantly reduced by 80 % and 81 %, respectively. The remarkable hemostatic effect of the PAAL hydrogel is attributed to the presence of carboxyl groups on its surface. These carboxyl, dynamic disulfide, and ester groups can establish various non-covalent internal and hydrophobic interactions with the polar groups on the wetted surfaces of the liver and tail, facilitating adhesion. Consequently, this interaction enables instantaneous hemostasis and the formation of a robust hemostatic barrier.

Conformal adhesion properties arise from the ability to conform tightly to complex surfaces while maintaining stable adhesion in dynamic environments. Achieving conformal adhesion to wet and dynamic biological tissues, such as heart and brain tissues, presents a significant challenge for medical dressings and implantable devices. Flexible electronics must seamlessly conform to irregular surfaces or curved substrates to enable precise signal acquisition. Conventional rigid materials struggle to adapt to dynamic deformation. Fig. 5G illustrates commercial silver chloride electrodes (Ag/AgCl), PAA hydrogel, and PAAL hydrogel laminated onto a model of pigskin with folds. The rigid commercial Ag/AgCl electrodes exhibited noticeable gaps when in contact with the porcine skin surface. The PAA hydrogel also failed to conform adequately, leaving various gaps, both large and small, when pressed against the uneven skin surface. In contrast, the PAAL hydrogel demonstrated superior adhesion to the wrinkled skin, forming a tight bond with the bumpy surface. We utilized PAAL hydrogels with printed electrodes under mild pressure and conducted peeling tests to evaluate their conformal contact and adhesion on ex vivo brain tissue in the presence of tissue fluid and blood. As shown in Fig. 5H, the hydrogel exhibited clear conformal fitting to the wrinkled brain surface and demonstrated strong adhesion, evidenced by its ability to lift the surface tissue during tweezer-assisted peeling.

To better demonstrate the effect of conformal adhesion, as illustrated in Fig. 5I, we tested the lateral contact and adhesion of commercial electrodes, PAA hydrogels and PAAL hydrogels on rough shaped polymer surfaces. The sheets of commercial electrodes and PAA hydrogels exhibited gaps at the interface, whereas the PAAL hydrogels with rough surfaces displayed a reduced contact area, particularly at wrinkles and depressions where a tight fit was observed with minimal gaps. Next, we placed it in the arm muscle position and significant EMG signals could be detected (Fig. S20, Supporting Information). Notably, the PAAL hydrogel exhibits rapid cohesion, thereby generating strong anchoring forces at the dynamically wetted soft tissue adhesion interface. This phenomenon arises from the close modulus matching between the hydrogel and brain tissue, which mitigates interfacial delamination caused by mechanical mismatch and enhances adhesion stability (Fig. 5J). Upon contact, the hydrogel can partially conform to the microscale roughness of brain tissue, forming mechanical interlocking that further strengthens the physical and mechanical anchoring effect and enables excellent adhesion performance on moist, compliant brain surfaces. In addition, we quantitatively evaluated the hydrogel's adhesion on wet, irregularly curved surfaces. The results show that the shear adhesion strength of PAAL hydrogels on hydrated brain tissue increases significantly with prolonged pressing time, reaching a maximum peeling strength of 16.3 kPa within 30 min, surpassing that of most commercial tissue adhesives and further confirming its robust interfacial integration capability (Fig. 5K). PAAL hydrogel was also able to maintain a high-quality retention of 78 % after 48 h of placement at room temperature, demonstrating its environmental stability under practical applications (Fig. S21, Supporting Information). Self-healing properties are important for the lifetime of hydrogel electrodes. Due to the fast dynamic exchange properties of the lipoic acid matrix and multiple dynamic bonding networks (including hydrogen bonding and dynamic disulfide bonding), the PAAL hydrogel have great potential for excellent self-healing properties. Specifically, we conducted a series of self-healing performance studies. As shown in Fig. S22 (Supporting Information), at room temperature, the scratches under the surface scratches healed automatically within 4 h. The circularly stained samples cut in the middle could be rapidly healed automatically at room temperature and were able to be stretched, which demonstrated the self-healing ability of PAAL hydrogel at room temperature (Figs. S23 and S24, Supporting Information). It is worth noting that the self-healing properties of PAAL hydrogels within 2 h after fracture were demonstrated by tensile experiments, which showed that the tensile strength reached 80 % of that of the initial sample after 2 h (Fig. S25, Supporting Information), Similarly, the Young's modulus showed a surprising self-healing effect (Fig. S26, Supporting Information), which demonstrates the strong self-healing ability of PAAL hydrogels in a short period of time.

### 2.5 Evaluation of cytocompatibility, antimicrobial, cell migration and angiogenesis

**Fig. 6:**
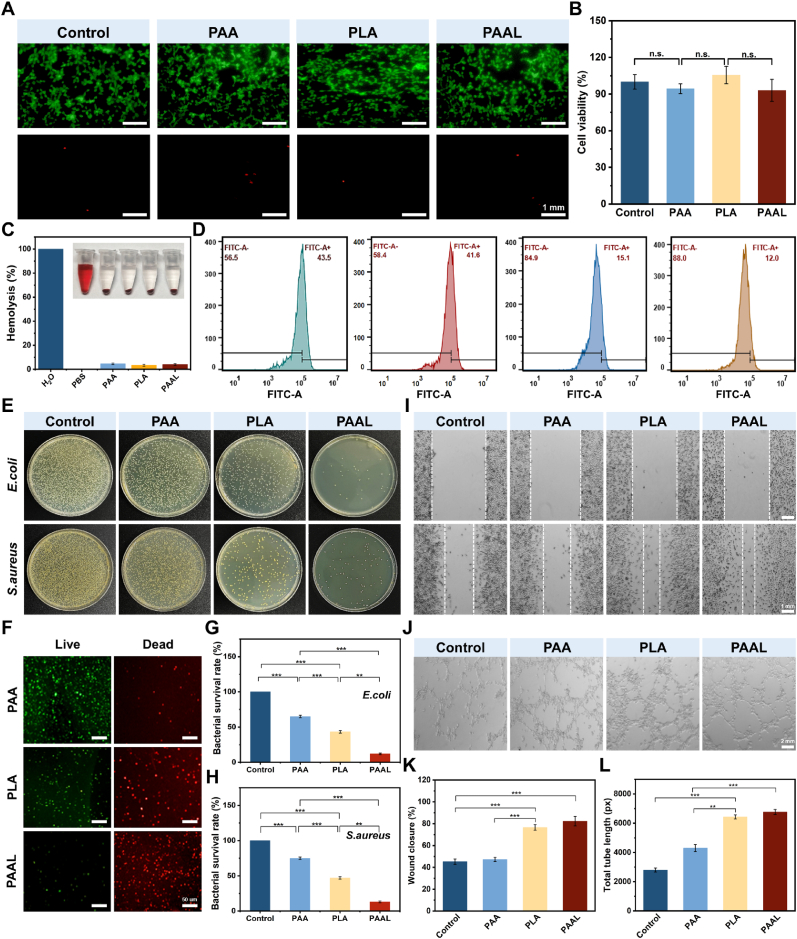
Biocompatibility and in vitro antimicrobial activity.A) Live/dead staining images of HUVEC cells after different hydrogel cultures. B) Survival of HUVEC cells after hydrogel treatment. C) Hemolysis rates of hydrogels. D) Intracellular ROS levels. E) Killing images of bacterial coatings by different hydrogels. F) Bacterial live-dead staining images. G) Survival of *E. coli* after co-culture with different hydrogels. H) Survival of *S. aureus* after co-culture with different hydrogels. I) Scratch assay assessing the proliferation and migration abilities of L929 cells. J) Images of tube formation by HUVEC after coincubation with hydrogel extracts for 6 h. K) Quantification of wound closure ratios in the scratch assay. L) Tube length for tube formation test, ∗p ≤ 0.05, ∗∗p ≤ 0.01, and ∗∗∗p ≤ 0.001.

Good biocompatibility is a crucial indicator for in vivo applications. In this regard, we investigated the biocompatibility of PAAL hydrogel [[43], [44], [45]]. First, we conducted a toxicity test on cells in vitro. Live/dead cell staining revealed that the majority of cells were viable and morphologically intact, with only a few dead cells exhibiting red fluorescence (Fig. 6A). The results demonstrated that human umbilical vein endothelial cells (HUVECs) co-cultured with PAA, PLA, and PAAL hydrogels maintained over 90 % viability across all hydrogel groups (Fig. 6B). Similarly, PAAL hydrogel exhibited a 94 % survival rate for L929 cells (Figs. S27 and S28, Supporting Information), indicating that PAAL hydrogel possesses good biocompatibility. Furthermore, the hemolysis rate for both the blank hydrogel and PAAL hydrogel was less than 5 % (Fig. 6C).

Reactive oxygen species (ROS) are detrimental factors that provoke excessive inflammation. We utilized the DCFH-DA probe to evaluate intracellular ROS levels and observed that the fluorescence intensity in both the PAA and control groups was identical, suggesting that PAA does not scavenge ROS. Conversely, CLSM images revealed a reduction in fluorescence intensity in both the PLA and PAAL groups. These results underscore the significant role of LA in ROS clearance [46]. To further quantify the efficacy of ROS scavenging, we employed flow cytometry (Fig. 6D). Compared to the control group, the fluorescence positivity rates in the PLA and PAAL groups decreased from 43.5 % to 15.1 % and 12.0 %, respectively. Therefore, the PLA and PAAL groups exhibited a robust capacity for ROS elimination.

Bacterial infections are also a common complication of wounds and can significantly impede the healing process [47,48]. We conducted antimicrobial tests against *Escherichia coli* (*E. coli*) and *Staphylococcus aureus* (*S. aureus*) using the plate counting method. As illustrated in Fig. 6E–H, the number of *E. coli* and *S. aureus* in the PAA group was comparable to that of the control group. Notably, the antimicrobial efficacy against *E. coli* and *S. aureus* was markedly enhanced, with an inhibition rate of up to 90 % after co-incubation with the PAAL hydrogel group, attributed to the presence of AgNPs. The antimicrobial activity of the PAAL hydrogel results from the synergistic effects of LA-derived active molecules and AgNPs. The LA-derived molecules contribute a moderate antibacterial effect, whereas the incorporation of AgNPs accounts for the markedly enhanced inhibition observed in PAAL, thereby strengthening the overall antimicrobial performance. These small molecules effectively disrupt the bacterial cell membrane and increase its permeability. Additionally, AgNPs disrupt the bacterial cell membrane, and the combined action of LA-based molecules and AgNPs provides a comprehensive antimicrobial effect.

In addition, neovascularization and cell proliferation play a crucial role in promoting granulation tissue formation, thereby accelerating wound healing. Fig. 6I shows the migratory behavior of L929 cells cultured in various hydrogels. The wound scratch assay demonstrated that the PAAL hydrogel group significantly enhanced cell migration, with both cell proliferation and migration markedly improved in the PAAL hydrogel group compared to the control group. Subsequently, we conducted a tube formation assay to evaluate the potential of PAAL hydrogels to promote angiogenesis in vitro. Fig. 6J presents a representative image of the capillary-like structures formed by HUVECs treated with the PAAL hydrogel. Compared to the control group, the capillaries in the PAAL hydrogel group exhibited greater completeness and branching. Specifically, as shown in Fig. 6K and L, the unhealed area in the PAAL hydrogel group was reduced by 37.5 % compared to the control group, indicating that the PAAL hydrogel promoted cell proliferation and migration. Furthermore, the total branch length of the PAAL hydrogel group was significantly increased, demonstrating its strong pro-angiogenic potential in vitro.

### 2.6 Implantable bioelectronics

**Fig. 7:**
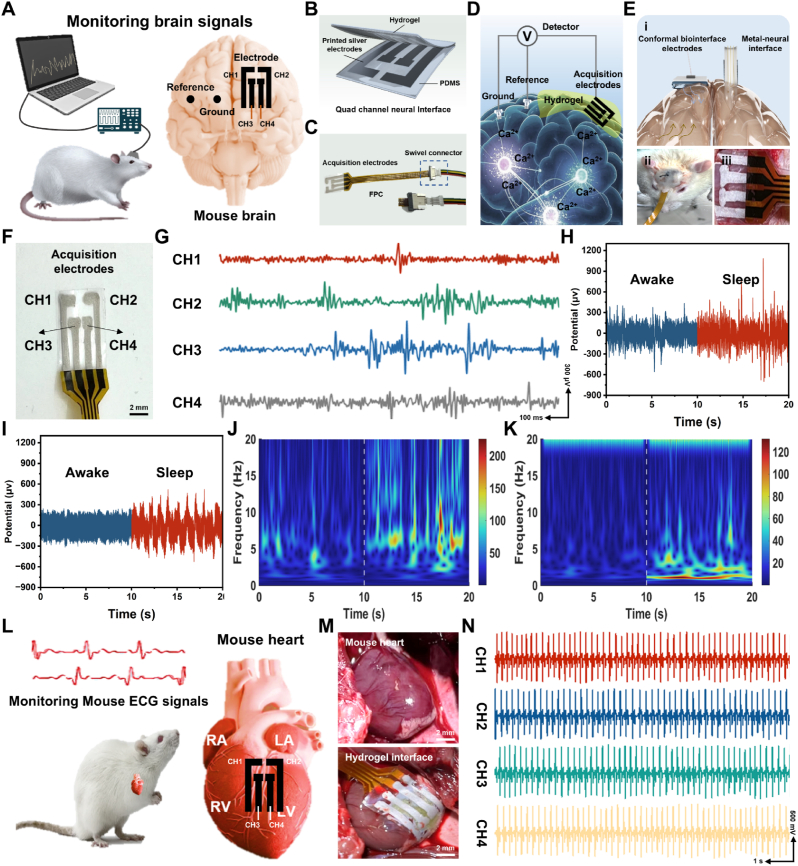
A) Illustration of the connection of rat ECoG recordings. B) Illustration of PAAL hydrogel assembled into a four-channel monitoring electrode. C) Image of connection based on PAAL hydrogel neural interface. D) Schematic diagram of the detection device used for ECoG recording. E) Image of a viscoelastic array of PAAL hydrogel neural interfaces placed on the surface of rat cerebral cortex. F) Image of PAAL hydrogel electrode interfaces with the channels named CH1, CH2, CH3, and CH4. G) Typical ECoG signals recorded through 4 channels. H) ECoG signals of the rat recorded with commercial electrodes. I) ECoG signals of the rat recorded with PAAL hydrogel. J) Signal spectrum of Fig. 7H. K) Signal spectrum of Fig. 7I. L) Illustration of the connection of rat ECG recordings. M) Image of a viscoelastic array of PAAL hydrogel neural interfaces placed on the surface of rat heart. N) Typical ECG signals recorded through four channels.

ECoG measures local field potentials generated by neurons in the cerebral cortex, enabling electrophysiological recordings with high spatial and temporal resolution across extensive brain regions (Fig. 7A) [[49], [50], [51]]. We validated the efficacy of PAAL hydrogel as a bioelectronic interface device for recording surface potential signals from the rat cerebral cortex. Prior to implantation, the PAAL hydrogel was assembled, specifically, four-channel electrodes were patterned on a PDMS substrate, and the hydrogel was covered with a surface that was assembled into conformal hydrogel-biointerface electrodes (CHBE). The adhesive layer, electrode layer, and PDMS encapsulation layer are included, as illustrated in Fig. 7B. Given that the modulus of the hydrogel closely matches that of brain tissue, bonding the PAAL hydrogel neuroelectrodes to the surface of the cerebral cortex facilitates an infiltration-bonding process with the tissue fluid, resulting in a conformal contact between the neuroelectrodes and the cerebral cortex. The conformal attachment of the PAAL hydrogel nerve electrode enables the recording of ECoG signals from the surface of brain tissue, thereby preventing the physical damage associated with the penetration of rigid electrodes. Furthermore, the high transparency of the PAAL hydrogel allows for clear observation of the relative positions of the electrodes through the hydrogel layer, which facilitates the accurate determination of the attachment site, and the introduction of ionic liquids and metal ions endow the hydrogel with good electrical conductivity, which facilitates signaling (Fig. S29, Supporting Information). ECoG signal recording experiments were conducted using an electrophysiology system (Fig. 7C and D). The electrode arrays were placed on the dura mater of rats. All four electrode channels successfully recorded electrical activity (Fig. 7E and F), and representative sequences of recorded ECoG signals were obtained (Fig. 7G). The integrated electrodes within the array measured local field potentials generated by cortical neurons through the conformal contact of the adhesive layer with the cerebral cortex surface. For comparative analysis, we also employed metallic rigid electrodes (stainless steel) as a control.

The ECoG signal values recorded during the sleep state exhibited significantly higher amplitude and power compared to those recorded in the awake state. This phenomenon may be attributed to the synchronization of a large number of neurons discharging and the synaptic resetting process that occurs during sleep [52]. Notably, as illustrated in Fig. 7H and I, the ECoG signals recorded by the two probes were similar, despite the conductivity of the metal electrode being significantly higher than that of the PAAL hydrogel. This similarity can be explained by the potential impedance mismatch and mechanical incompatibility at the contact interface between the rigid electrode and the tissue, which results in signal quality degradation. In contrast, the hydrogel forms closer contact with the brain tissue, thereby reducing interface impedance and facilitating more efficient signal transmission, ultimately leading to a more stable signal (Fig. 7J and K). To quantitatively evaluate this effect, we calculated the signal-to-noise ratio (SNR) of the ECoG recordings in the delta (0.5–4 Hz) and theta (4–8 Hz) ranges. In the awake state, these low-frequency components are relatively weak, and the SNR values obtained from the two electrode types were similar. In the sleep state, where the activity is much stronger, the CHBE electrodes exhibited markedly higher SNR than the commercial electrodes, reflecting the improved electrode-tissue coupling provided by the conformal hydrogel interface (Fig. S30, Supporting Information). In addition, the stable signal transmission has the potential to be used as flexible sensors in vitro [53]. We placed the hydrogels in different motion joints of the human body and generated repeated stable electrical signals through joint movements, which proved their promise as motion sensors, and we used them as temperature sensors which still possessed stable signal transmission (Figs. S31 and S32, Supporting Information), while repeated stretching at multiple fixed strains showed surprising signal stability (Fig. S33, Supporting Information).

To study the long-term performance of the rats, ECoG signal was recorded by CHBE for 4 weeks. Figs. S34 and S35 (Supporting Information) shows the changes in ECoG signal over time during sleep. We found that the intensity increased over time, but the signal was relatively stable. To verify the long-term biocompatibility of CHBE electrodes during ECoG recording, a sham-operated group was used as the control under the same conditions. Four weeks after implantation, histological assessments including H&E staining, Masson's trichrome staining and immunohistochemistry (IHC) were performed to examine brain tissue responses (Fig. S36A, Supporting Information). H&E staining showed uniform and clear morphology in the CHBE group, indicating a mild inflammatory reaction. Quantitative analysis of fibrotic thickness (Fig. S36B, Supporting Information) showed minimal tissue deformation and low fibrosis in the CHBE group (28 μm), similar to the sham group. IHC analysis was conducted to assess immune cell infiltration at the neural interface using fluorescence markers for nuclei (DAPI), astrocytes (GFAP) and microglia (IBA-1), followed by quantitative evaluation of cortical structural changes (Fig. S36C–D, Supporting Information). The CHBE group showed only a slight increase compared with the sham group. These results demonstrate that CHBE exhibits good biocompatibility and tissue tolerance, enabling stable and long-term ECoG signal recording at neural interfaces. Subsequently, we implanted PAAL hydrogel electrode arrays onto the rat heart (Fig. 7L), securing them in place through surface tension and plastic deformation as the organ continued to beat. Representative electrocardiograms (ECG; Fig. 7M and N) were recorded across all four channels. Remarkably, the electrode arrays demonstrated high signal stability and achieved a tight fit to the wetted heart surface, yielding realistic ECG signals in contrast to those obtained from rigid commercial electrodes (Figs. S37–38, Supporting Information). In addition, flexible electronics and electrodes are usually disposable and non-degradable, which puts a huge strain on the environment, and reusable flexible electrodes are essential for sustainable chemistry. To recover the PAAL hydrogel, it was immersed in 0.5 M NaOH solution. As shown in Fig. S39 (Supplementary Information), rapid degradation of the PAAL hydrogel was observed, with the sample gradually degrading within 60 min. It was demonstrated to possess excellent degradability and environmental friendliness.

## 3 Conclusion

In this study, we present a multifunctional polyacrylic acid/poly(alpha-lipoic acid)-NHS (PAAL) dual-network hydrogel based on dynamic covalent bonding. This hydrogel integrates temperature-responsive on-demand de-adhesion, conformal adhesion to wetted tissues, highly efficient self-healing, and excellent biocompatibility, offering a promising solution for complex biomedical applications. By combining dynamic disulfide bonding and dynamic exchange of poly(lipoic acid) chains with the hydrogen bonding network formed by the carboxylic acid groups of PAA, along with leveraging the interfacial reactivity of NHS esters for mechanical anchoring, the hydrogel exhibits strong adhesion (53.4 kPa) and low swelling (<10 %) under wet conditions. Furthermore, it enables rapid, non-invasive debonding triggered by mild temperature changes (5 °C). The PAAL hydrogel significantly accelerates hemostasis in both liver and tail bleeding models and adaptively adheres to complex, wet biological surfaces, including curved organs such as the heart and brain. In addition, its electrical conductivity and biocompatibility allow it to function as a conformal bioelectrode, seamlessly integrating with the brain cortex or myocardium for stable in vivo ECoG/ECG signal recording. This study presents a novel strategy for developing smart materials for hemostasis, organ and tissue repair, and implantable bioelectronics.

## 4 Experimental section

**Materials:** Lipoic acid (LA, 99 %), Tris(hydroxymethyl)aminomethane (Tris) were purchased from Sigma-Aldrich. N-hydroxysulfosuccinimide (NHS) and N,N′-methylenebisacrylamide (MBA) were purchased from Bide Pharmatech Ltd. Acrylic acid (AA), AgNPs (120 nm) and APS were purchased from Macklin. Deuterated solvents (DMSO-*d*_6_) and 1-vinyl-3-butylimidazolium tetrafluoroborate (VBIMBF_4_) were purchased from Sigma-Aldrich. All the reagents were used without further purification.

**Preparation of PAAL hydrogel:** 3 g of LA powder was dissolved in a Tris aqueous solution (50 wt%, 5 mL) with continuous stirring until the solution was clarified, then 1 g of LA-NHS powder (the preparation of which is listed in the Supporting Information) was added to this solution, and the resultant solution was stirred for 30 min, followed by the addition of 3 mL of AA and 200 μL of VBIMBF_4_ (0.1 mol L^−1^), Subsequently, 0.1 g of AgNPs was added with continuous stirring. and the addition of MBA (3 mg) and APS (30 mg) to this aqueous solution. MBA (3 mg) and APS (20 mg) were added to this aqueous solution and then injected into homemade PTFE molds, degassed under vacuum and polymerized at 70 °C for 4 h. The corresponding PAAL hydrogels were obtained, and for the sake of comparison, the PLA hydrogels were prepared by dissolving 3 g of LA and 1 g of LA-NHS powders in an aqueous solution of Tris, and pouring them into homemade molds for heat Polymerization. A pure PAA hydrogel was similarly prepared.

**Preparation of conformal hydrogel-biointerface electrodes (CHBE):** The hydrogel precursor was cast on a flexible microcircuit consisting of a conductive silver paste patterned layer and a PDMS encapsulated layer. The integrated CHBE was obtained after the hydrogel was triggered to solidify.

**Characterization:** FT-IR spectra of dried adhesive samples were tested using an FTIR instrument (Bruker, Germany). The spectral range of the tested wave numbers ranged from 4000 to 400 cm^−1^. Evaluations were performed using a rotational rheometer (Anton Paar, Austria) determine the storage modulus (G′) and loss modulus (G″). Samples dissolved in DMSO-*d*_6_ were subjected to proton nuclear magnetic resonance (^1^H NMR) spectroscopy using a spectrometer (Bruker, Germany). The contact angle of the hydrogel samples was evaluated at room temperature using a contact angle meter (HARKE-SPCA, China). Different regions of each sample were measured at least three times, each time using 4 μL of distilled water. Using a Rigaku Smart Laboratory X-ray diffractometer (Rigaku, Japan). The crystal structure was characterized by powder X-ray diffraction (XRD) analysis and scanning electron microscope (FE-SEM, HITACHI Regulus 8100) at an accelerating voltage of 5 kV.

**Adhesion performance experiments:** The adhesion strength of the hydrogels was tested using a tensile tester (Instron 5567) by applying hydrogels with a bonding area of 25 mm × 25 mm to different substrates. The hydrogels were pulled to failure at a tensile speed of 100 mm/min. The adhesion strength was calculated by dividing the maximum load by the initial bonding area. The wet adhesion strength of hydrogels to isolated organ tissues was also measured using fresh porcine muscle, fresh porcine heart, stomach and liver as representative substrates. Lap shear tensile tests were performed at a rate of 50 mm/min until failure testing. Similarly, the adhesion strength of PAAL hydrogel was evaluated in different liquid environments including seawater, PH = 3 and 1 M NaCl. And tested at different tensile speeds (25–125 mm/min).

**Method of Burst Pressure Test:** Using a hole punch, cut round holes (5 mm diameter) in the center of the pigskin. A hydrogel patch of 15 mm diameter was then pressed onto the round hole in the pigskin (epidermal layer). And it was placed under 500 g weight for 1 min and clamped in the middle of a homemade bursting pressure test device and then the rupture strength was measured. The device was filled at a constant rate via an air pump until the hydrogel was burst by air pressure from the pigskin notch and the maximum pressure was recorded using a manometer. Three replications of each experiment were performed.

**Ex-Vivo Organ Sealing Experiment:** The porcine stomachs were rinsed with excess PBS, and the stomachs were subjected to copious water filling until they were full, and a 5-mm notch was made with a knife. Observe its obvious continuous leakage of water from the notch for a more visual observation. Then, PAAL hydrogel was applied to the notch and pressed gently for 30 s. The leak tightness of the porcine stomachs was then investigated by observing the closure of the leakage ports to see if water flowed out. Similarly, the porcine intestine and porcine bladder were subjected to the same study.

## CRediT authorship contribution statement

**Zhenchun Li:** Writing – original draft, Investigation, Data curation, Conceptualization. **Tiantian Li:** Writing – original draft, Validation, Methodology, Investigation. **Rongfeng Ge:** Validation, Supervision, Methodology. **Feixiong Chen:** Writing – review & editing, Resources, Funding acquisition. **Chuang Du:** Writing – original draft, Supervision, Resources, Investigation. **Dongxu Wang:** Writing – original draft, Supervision, Resources, Project administration. **Lei Wang:** Writing – review & editing, Supervision, Project administration, Funding acquisition, Conceptualization.

## Animal ethics statement

All animal experiments were conducted in accordance with the protocol (Protocol No. 2025043001) approved by the Institutional Review Board of Jilin University, and in compliance with relevant institutional guidelines and laws. Adult male SD rats (250–300 g) and adult male mice (30–35 g) were purchased from Liaoning Changsheng Biotechnology Co., Ltd. During the experiment, all rats and mice were housed under controlled conditions and provided with free access to food and water.

## Declaration of competing interest

The authors declare that they have no known competing financial interests or personal relationships that could have appeared to influence the work reported in this paper.

## Data Availability

Data will be made available on request.
