# Supplementary material for: Thermo-responsive, on-demand adhesive and tissue-conformal hydrogel electrodes for organ repair and brain-computer interfaces
Source: Mater Today Bio. 2025 Dec 20;36:102705. doi: 10.1016/j.mtbio.2025.102705 (PMC12813216; doi:10.1016/j.mtbio.2025.102705)
Supplement: Multimedia component 1 [file mmc1.docx]

Supporting Information

**Thermo-Responsive, On-Demand Adhesive and Tissue-Conformal Hydrogel Electrodes for Organ Repair and Brain-Computer Interfaces**

*Zhenchun Li^a1^, Tiantian Li^b1^, Rongfeng Ge^a^, Feixiong Chen^c*^, Chuang Du^d*^, Dongxu Wang^b*^, and Lei Wang^a*^*

^a^ Key Laboratory of Molecular Enzymology and Engineering of Ministry of Education, School of Life Sciences, Jilin University, Changchun 130012, P.R. China
^b^ Laboratory Animal Center, College of Animal Science, Jilin University, Changchun 130062, P.R. China
^c^ Disease Networks Research Unit, Faculty of Biochemistry and Molecular Medicine, University of Oulu, 90014 Oulu, Finland
^d^ Changchun Institute of Applied Chemistry, Chinese Academy of Sciences, Changchun 130022, P.R. China
^1^ These authors contributed equally to this work.
^*^ Corresponding authors: Feixiong Chen (feixiong.chen@oulu.fi), Chuang Du (duch@ciac.ac.cn), Dongxu Wang (wang_dong_xu@jlu.edu.cn), and Lei Wang (w_lei@jlu.edu.cn)

**Supplementary Method**

**Cytocompatibility Test**

Extracts were prepared in a complete medium (30 mg/ml) at 37 °C for 24 hours and subsequently filtered through a 0.22 µm filter. Mouse fibroblast cells (L929) and Human umbilical vein endothelial cells (HUVEC) at a density of 1×10^4^ cells/well were seeded in 96-well plates and treated with 100 µl of leaching solution at concentrations of 30 mg/ml after cell adhesion (n = 5). Cell viability was assessed after 24 hours of culture using the CCK-8 assay, following the manufacturer's instructions (IV08-100, Invigentech, USA). Absorbance was measured at 450 nm, with the blank group representing the absorbance of the CCK-8 reagent and culture medium alone. Additionally, live/dead cell staining was performed utilizing the Calcein-AM/PI double staining kit (Beyotime, China), following the provided instructions. The cell viability was then examined using an OLYMPUS fluorescence microscope. Cell viability was calculated as follows:

$C\text{ell} viability (\%)=\frac{OD_{test}\text{-}OD_{blank}}{OD_{control}\text{-}OD_{blank}}\times100\text{\%}$(1)

where the $OD_{test}$, $OD_{blank}$, and $OD_{control}$ represent the absorbance values of the test, blank, and control groups at 450 nm, respectively.

**Hemolysis Assay**

The pulverized hydrogel samples were mixed with 2 mL of PBS to make a dispersion solution at a concentration of 10 mg/mL solution and then add 200 µL of RBC suspension. After 1 hour of incubation at 37 °C, the suspension was collected and centrifuged at 2000 rpm. The suspension was collected and centrifuged at 2000 rpm. Collect the suspension and centrifuge at 2000 rpm for 15 minutes. The supernatant was photographed and the absorbance at 540 nm was measured using a microplate reader. PBS and water were used as controls. The hemolysis rate was calculated using the following formula:

$Hemolysis ratio (\%)=\frac{A_{s}\text{-}A_{n}}{A_{p}\text{-}A_{n}}\times100\text{\%}$(2)

where the $A_{s}$, $A_{p}$, and $A_{n}$ are the absorbance values of the samples, positive control, and negative control, respectively.

**In Vitro Antimicrobial Assessment**

The antimicrobial activity of the hydrogel samples was tested by the ring of inhibition method. The samples were placed on medium containing bacteria (10^6^ CFU/mL) and cultured at 37 °C for 24 h. The antibacterial activity of the samples was assessed by the width of the circle of inhibition. The strength of the inhibitory activity was assessed by the width of the inhibitory circles on the hydrogel samples. The antimicrobial properties of the hydrogels were evaluated by plate spreading, live/dead bacterial staining and scanning electron microscopy imaging. 100 mg of hydrogel was co-incubated with 100 μL of bacterial suspension (10^6^ CFU/mL) at 37 °C for 4 h. Subsequently, 800 μL of PBS solution was added, and 10 μL of the suspension was evenly spread on LB agar plates. 18 h of incubation at 37 °C was followed by taking pictures of the plates and counting the colonies. 10 μL of bacterial suspension (10^6^ CFU/mL) in PBS (990 μL) was added as blank group. The antimicrobial rate was calculated as follows:

$Antibacterial ratio (\%)=\frac{C_{blank}\text{-}C_{sample}}{C_{blank}}\times100\text{\%}$(3)

where $C_{blank}$ and $C_{sample}$ are the colonies on the blank and sample groups, respectively. The antibacterial activity of the organohydrogels was further evaluated using bacterial live/dead staining. After co-culture with different hydrogels, the bacteria were co-stained with PI and DMAO for 30 min, rinsed through buffer for centrifugation. Images were taken using fluorescence microscopy.

**Scratch Wound Healing Assay**

The scratch wound healing assay for L929 cells was conducted in 6-well plates. Cells were seeded at a density of 2 × 10^5^ cells per well and cultured until they reached confluence. Once confluent, linear scratches were introduced into the cell monolayer using a sterile pipette tip. The hydrogel was then placed in the region and co-cultured with L929 cells. Images of the wound area were captured at 0 and 24 hours using a microscope. The wound closure rate was calculated as the ratio of the wound area that had closed at 24 hours to the initial wound area.

**In Vitro ROS Scavenging**

The experiment involved seeding L929 cells (5 × 10^5^ cells in 1 mL of DMEM) into a 6-well plate. After 12 h of cell adherence, each group received a 6 h-exposure to DMEM containing 300 nM H_2_O_2_. Subsequently, each group was treated with hydrogel extracts for 12 h. After rinsing three times with PBS, cells were treated with 10 μM 2′,7′-dichlorofluorescein diacetate (DCFH-DA) for 20 min. Intracellular ROS levels were assessed using a fluorescent microscope (Olympus IX71, Japan). For the flow cytometry analysis, L929 cells were treated similarly, and a cell suspension (2 × 10^5^ viable cells) was finally collected and analyzed with a BD LSR Fortessa flow cytometer.

**Tube Formation Assay**

Growth factor-reduced Matrigel was added to wells of a 96-well plate and solidified in an incubator for 30 min. HUVECs (2 × 10^4^ cells) were seeded onto the solidified Matrigel and co-cultured in high glucose DMEM supplemented with extracts of PAA, PLA and PAAL hydrogel or blank medium for 6 h. The tube formation was then observed under a microscope

**In Vivo Hemostatic Performance of PAAL Hydrogel**

The in vivo hemostatic performance of the PAAL hydrogel was evaluated using a mouse liver puncture bleeding model (mice, 30-35 g, male). According to the previously reported method, the mice were anesthetized with isoflurane and then secured onto a surgical corkboard. The rat's liver was exposed through an abdominal incision and the serous fluid around the liver was carefully removed. Pre-weighed filter paper was placed directly under the liver and separated from the abdominal incision by plastic wrap. Then a biopsy punch was used to make a liver tissue perforation with a diameter of 5 mm to establish a rat liver perforation model. Place the PAAL hydrogel and the hemostatic powder onto the wound separately. During the hemostasis process, record the amount of bleeding and the time taken to stop the bleeding. Each group contained 5 mice. All animal experiments were approved by the Institutional Review Board of Jilin University.

**Swelling Performance Test**

The PAA hydrogel and PAAL hydrogel were weighed and immersed in deionized water, with the change in weight observed and recorded at regular intervals. The swelling rate ($S\text{w}$) was calculated using the following formula:

$S\text{w}=(M-M_{0})/M_{0}\times100\%$(4)

where $M_{0}$ is the weight of the original PAA hydrogel and PAAL hydrogel and $M$ is the weight after water absorption.

**Motion Sensor Signal Recording**

The PAAL hydrogel sensor was connected to a Keithley 2750 digital source meter with wires pulled in order to monitor the movement of the body, insulating tape was tied to different parts of the volunteer. Changes in hydrogel resistance were recorded as the body performed a series of activities. The relative change in resistance. EMG signals were detected by a multichannel physiological signal acquisition system (RM6240EC). EMG signals were recorded by handshaking at intervals.

**ECoG Signal Recording**

Adult male SD rats (250-300 g) were used for ECoG recording. All procedures complied with relevant regulations. Rats were housed under a 12/12-h light/dark cycle for one week before surgery. Under sodium pentobarbital anesthesia, the scalp was exposed, subcutaneous tissue was bluntly separated, and a 5 × 5 mm cranial window was drilled to expose the dura.

Two stainless steel screws were implanted as reference and ground electrodes, and conformal hydrogel-biointerface electrodes (CHBE) were placed on the dura and fixed with dental cement. The monitored cortical area corresponded to the 25 mm^2^ craniotomy window. Rats were allowed to move freely during recording. ECoG signals were acquired using an RM6240EC multichannel system at 2 kHz and processed in MATLAB 2020a. Signals were band-pass filtered (0.5-100 Hz, FFT-based) and notch filtered at 50 Hz. No additional preprocessing was applied; segments with obvious artifacts were excluded by visual inspection.

Wake and sleep states were recorded in separate sessions. Behavioral scoring combined with video monitoring was used to classify states. Sleep was defined by closed eyes and immobility with occasional small twitches; wakefulness was defined by open eyes and spontaneous locomotor or exploratory activity. Recording began only after the animal had remained in a stable state for a continuous period. Three male SD rats were tested in each condition. For spectral analysis, power spectra were derived from a single artifact-free 10-s ECoG segment per state, and similar patterns were observed across individuals.

**ECG Signal Recording**

For implantation of conformal hydrogel-biointerface electrodes (CHBE), SD rats (male, weight 250-300 g) were selected to obtain ECG electrical signals. All animal experiments were performed in accordance with relevant laws and regulations. Before surgery, rats were kept in a 12/12-hour dark/light room for one week. First, the rats were anesthetized with sodium pentobarbital. The hair around the surgical area was shaved to expose the skin. Anesthetized with tribromoethanol and mechanically fixed, after general anesthesia, the rat's chest was opened through a thoracotomy in the third or fourth left intercostal area to expose the heart. The pericardium was removed from the rat heart using tiny forceps and most of the biological fluid was removed using gauze. The anterior surface of the heart was exposed during open-chest surgery, and a CHBE was gently pressed onto the ventricular surface to record signals, and a silver wire with a diameter of 0.5 mm was inserted subcutaneously into its leg as a reference electrode. And the ECG was recorded continuously.

**Supplementary Figure**


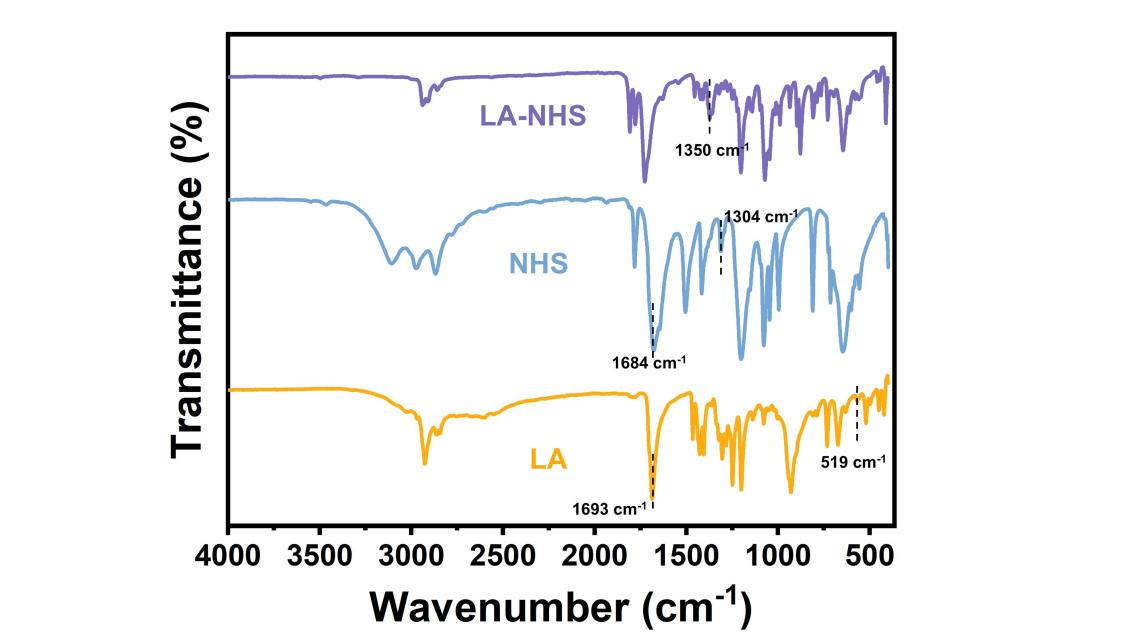


**Figure S1.** IR spectra of LA, NHS and LA-NHS.


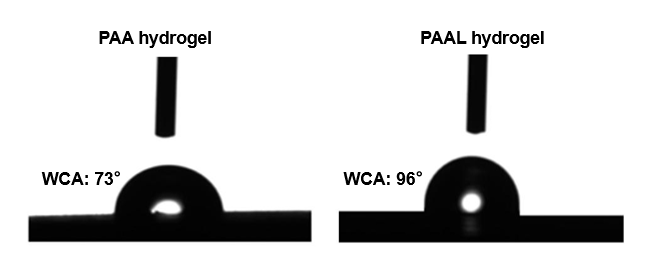


**Figure S2.** Static contact angle pictures of PAA hydrogel and PAAL hydrogel.


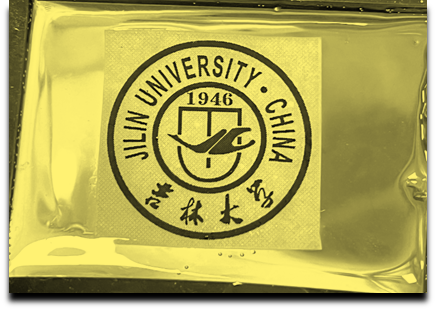


**Figure S3.** PAAL hydrogels prepared into thin slices behaved uniformly transparent and remained in their original state after being left at room temperature for 2 days.

**
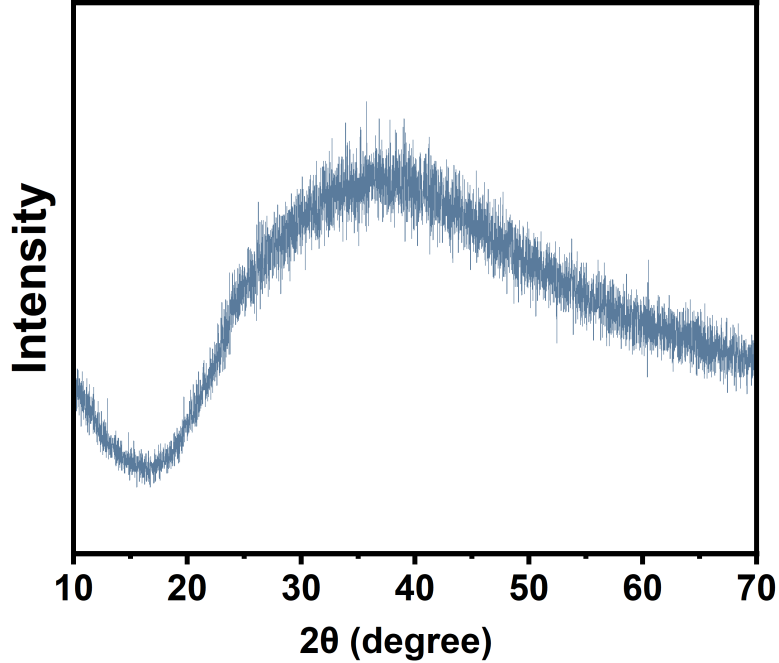
**

**Figure S4.** XRD spectra of PAAL hydrogel.

**
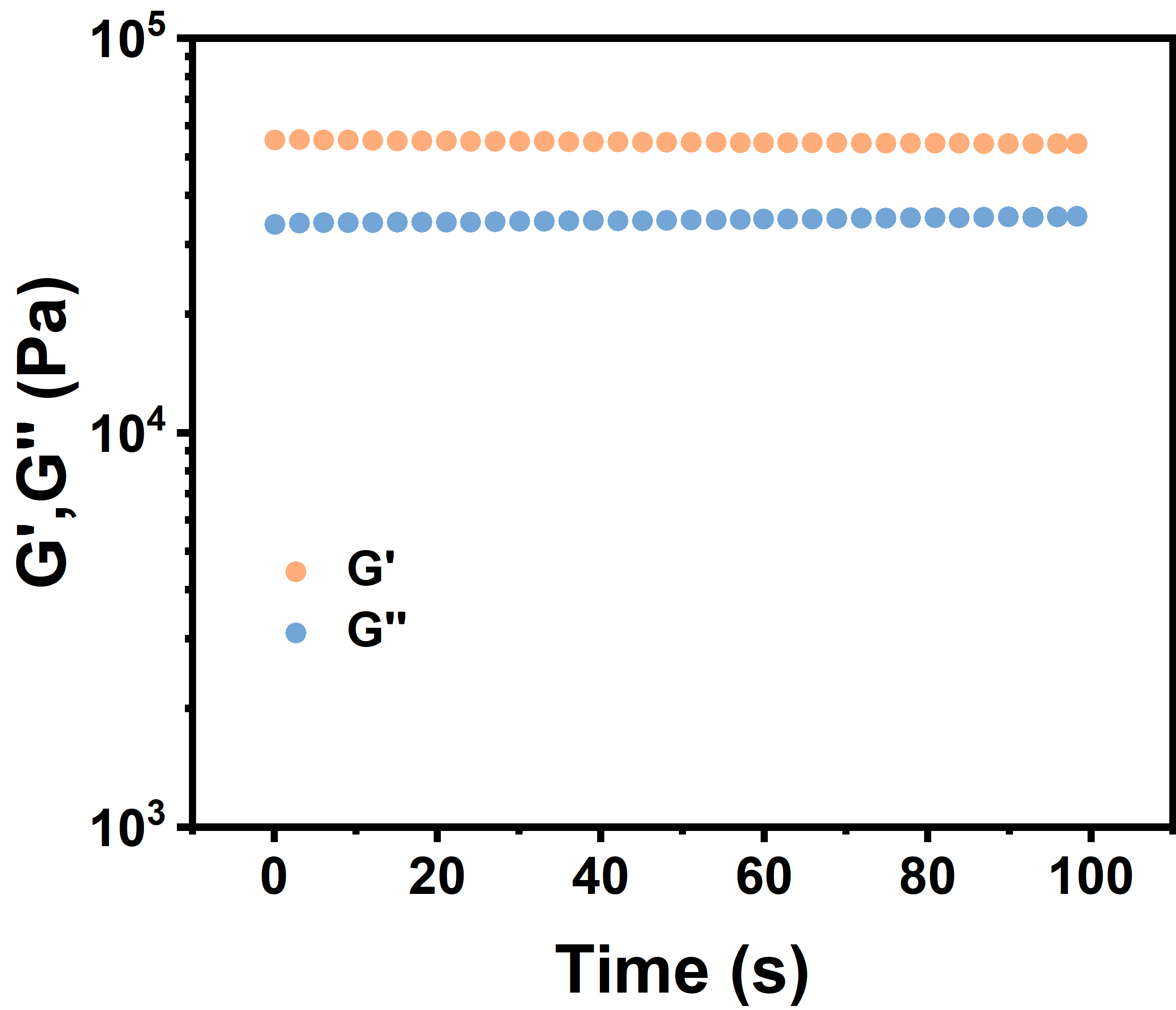
**

**Figure S5.** G′ and G′′ of PAAL hydrogel time scans.


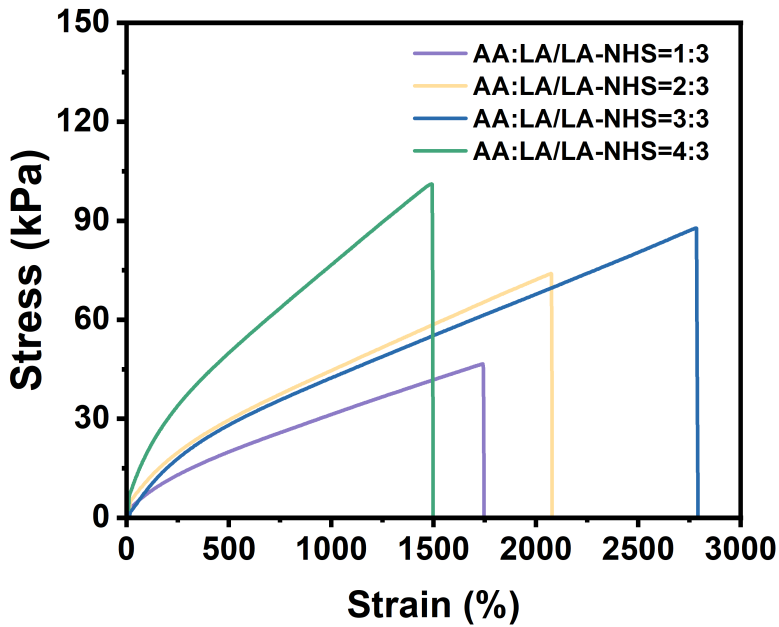


**Figure S6.** Tensile stress-strain curves of hydrogels prepared with different ratios of AA to LA/LA-NHS.


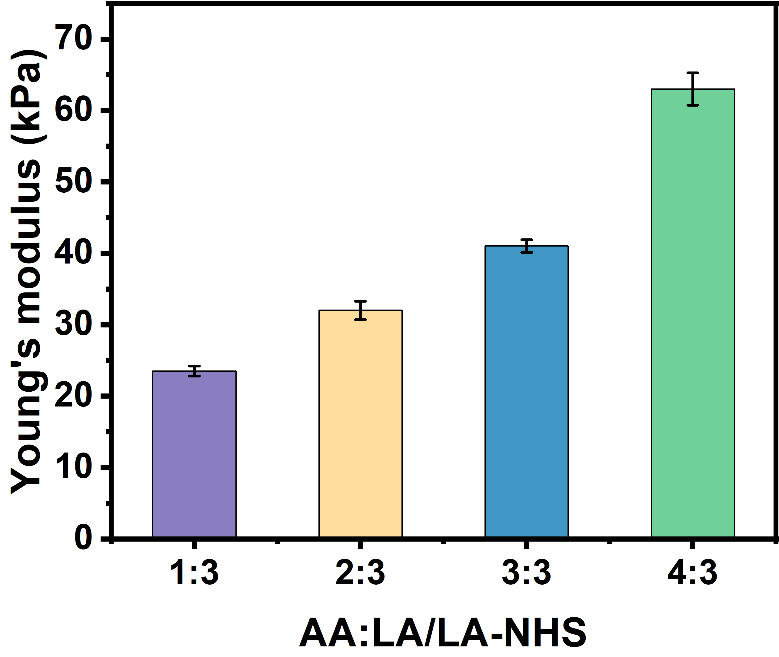


**Figure S7.** Young's modulus of hydrogels prepared with different ratios of AA to LA/LA-NHS.


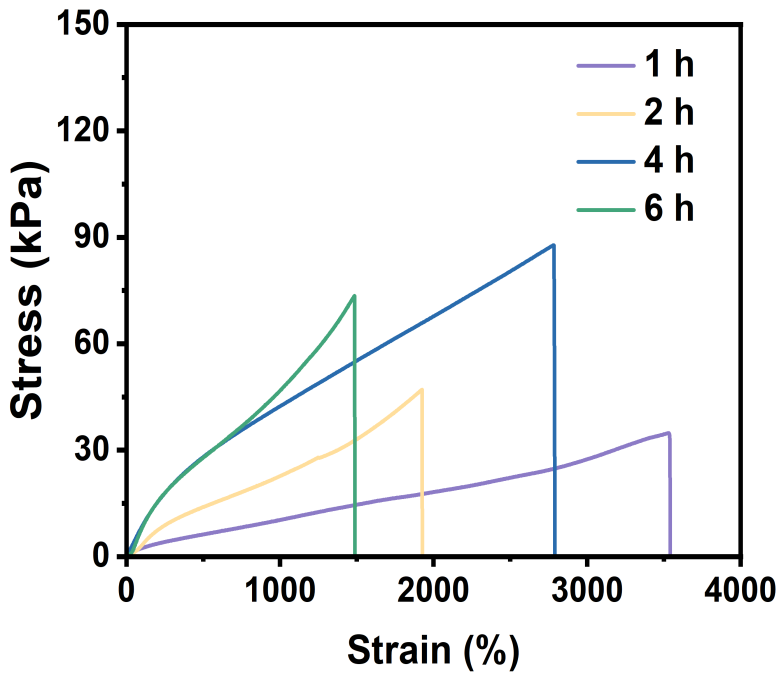


**Figure S8.** Stress-strain curves of hydrogels prepared with different holding heating times.


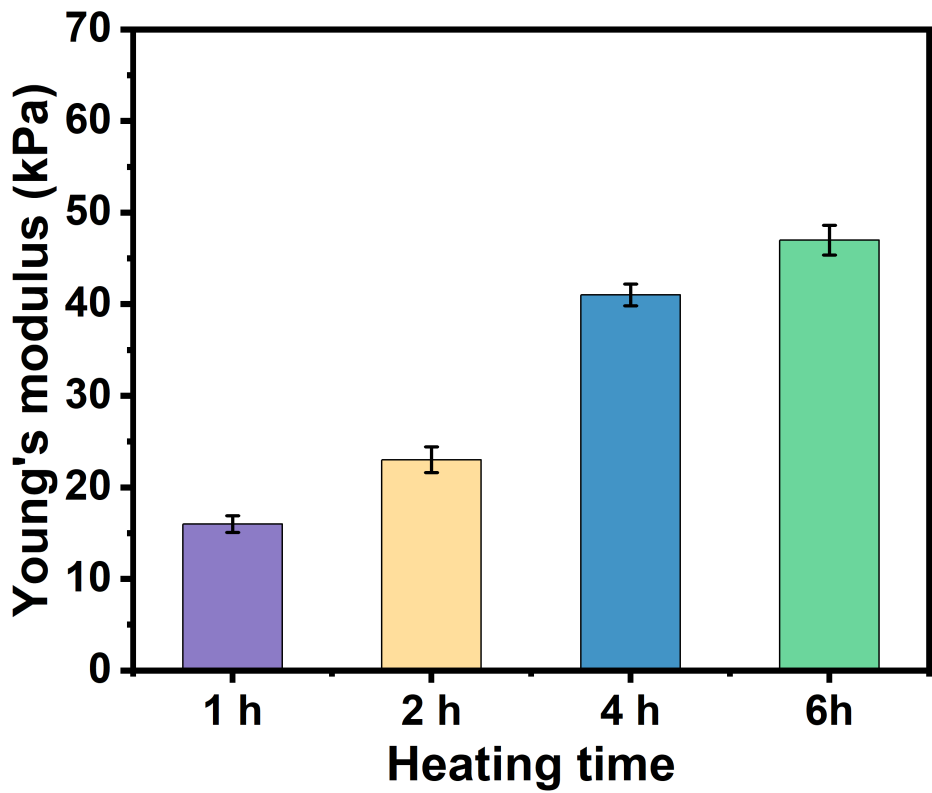


**Figure S9.** Young's modulus of hydrogels prepared with different holding heating times.


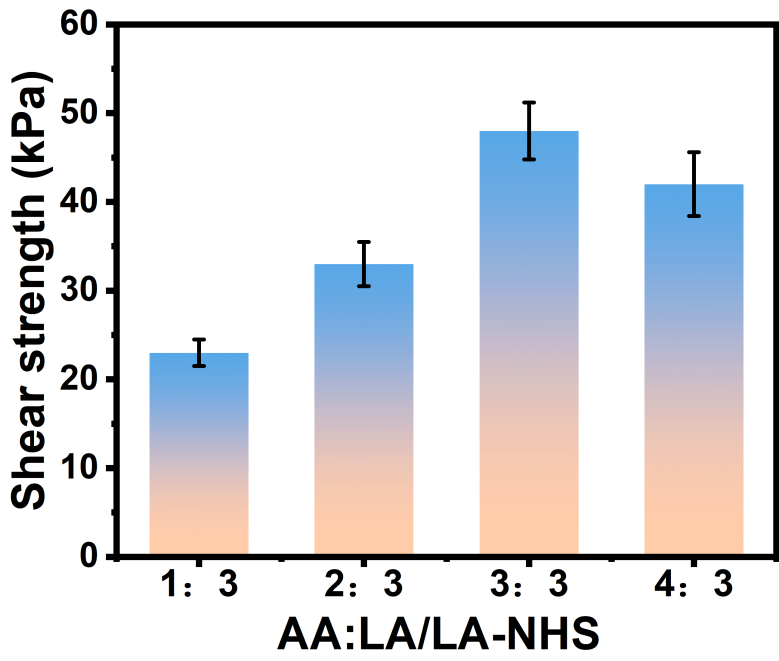


**Figure S10.** Shear strength of hydrogels prepared with different ratios of AA to LA/LA-NHS.


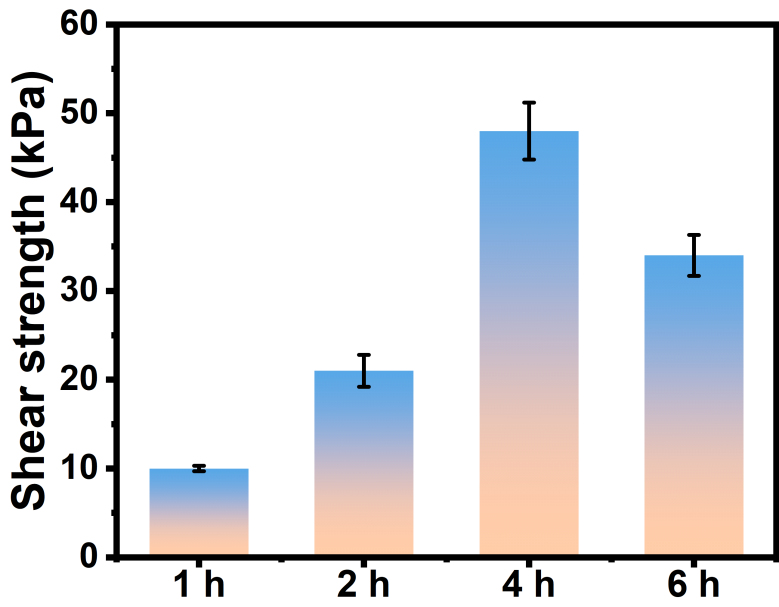


**Figure S11.** Shear strength of hydrogels prepared with different holding heating times.


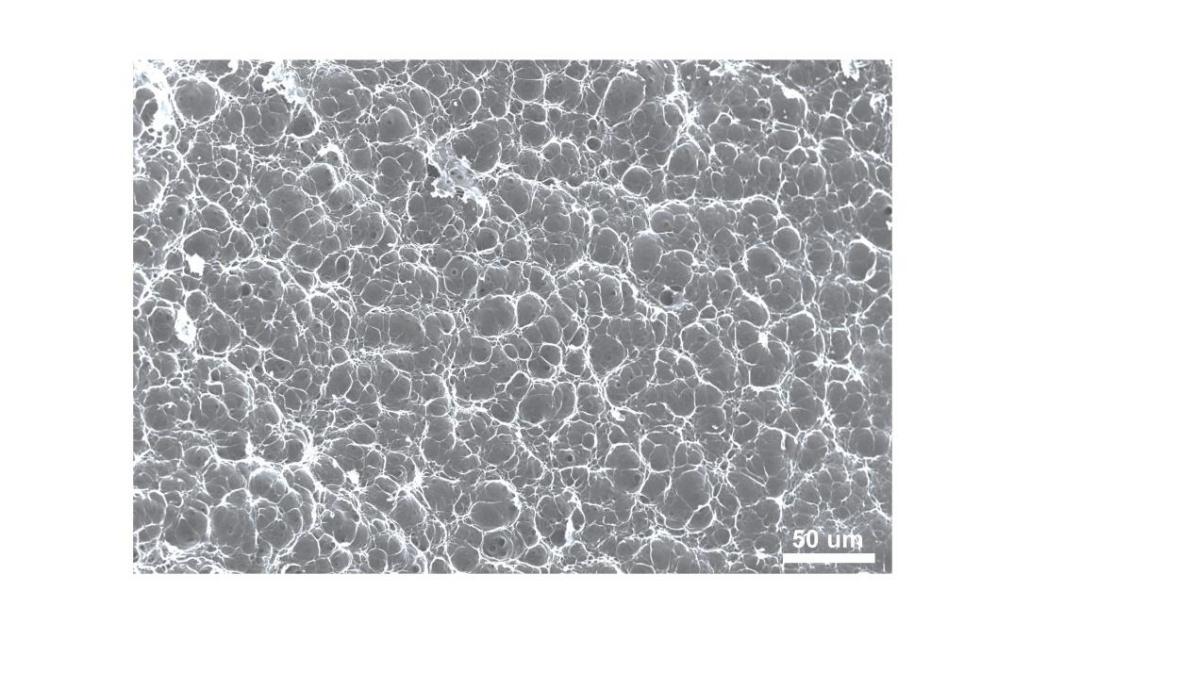


**Figure S12.** SEM results of PAAL hydrogels kept heated for 6 h.


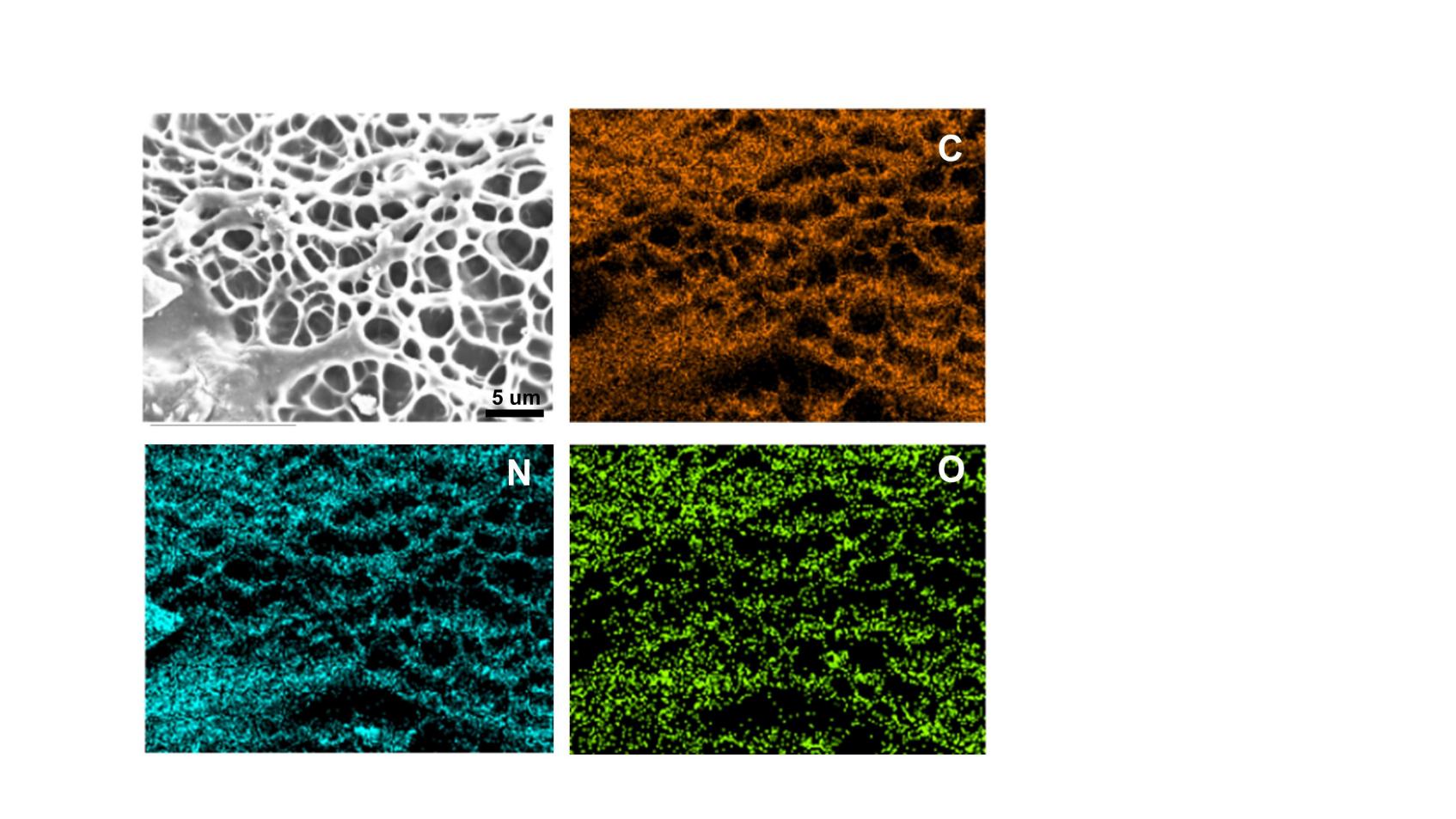


**Figure S13.** EDS results of PAAL hydrogels kept heated for 6 h.

| 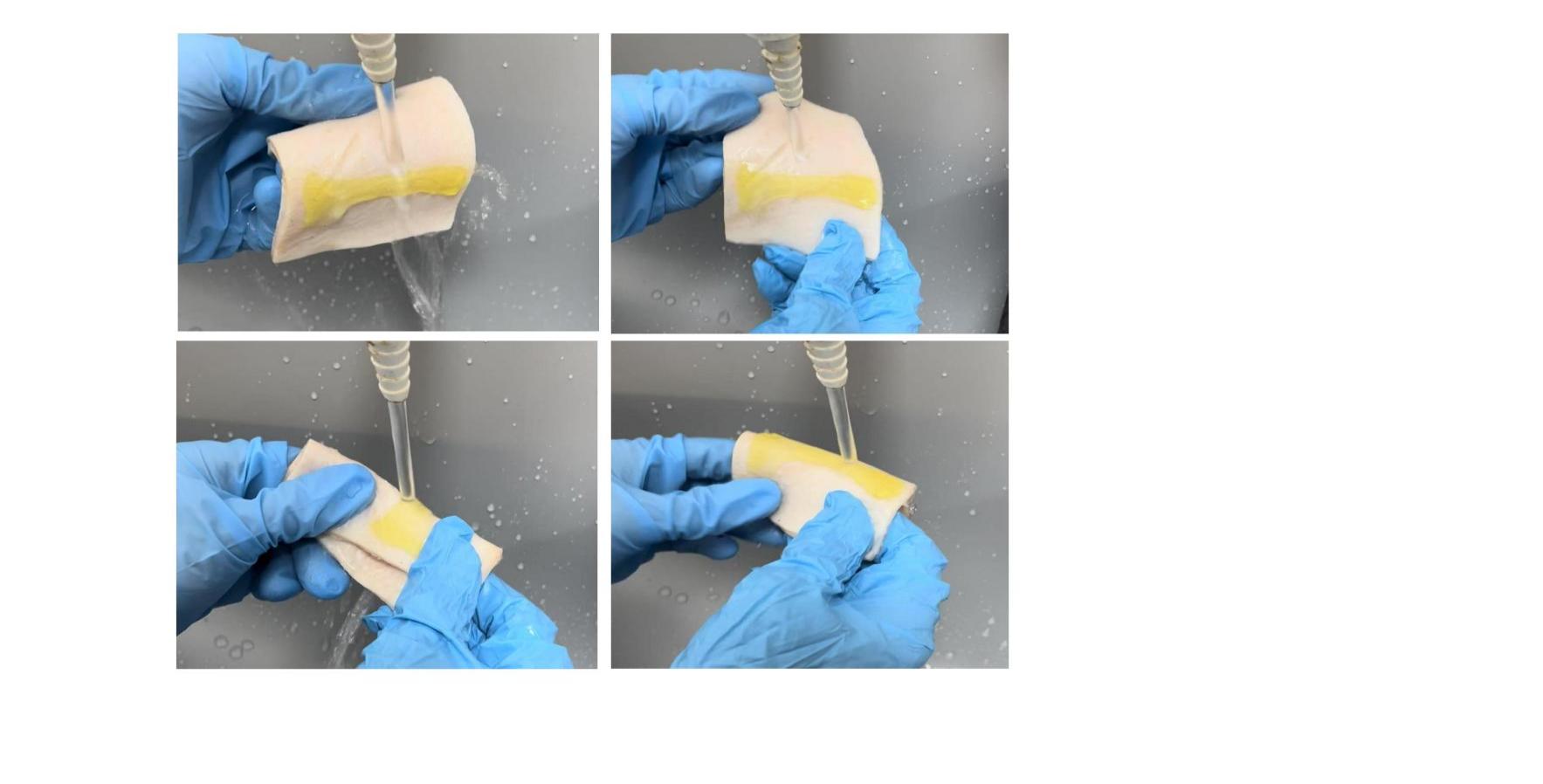 |
| --- |

**Figure S14.** PAAL hydrogel was adhered to moist pig skin and washed at all angles with a stream of water and remained firmly adhered.


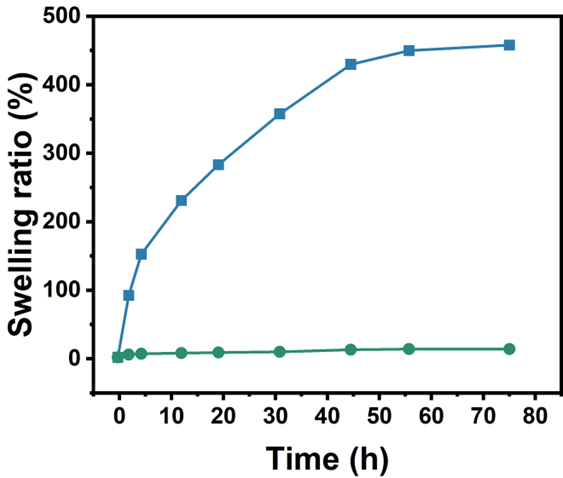


**Figure S15.** PAA hydrogel and PAAL hydrogel were tested for swelling rate in saline, and PLA hydrogel could not be measured for poor molding.

**
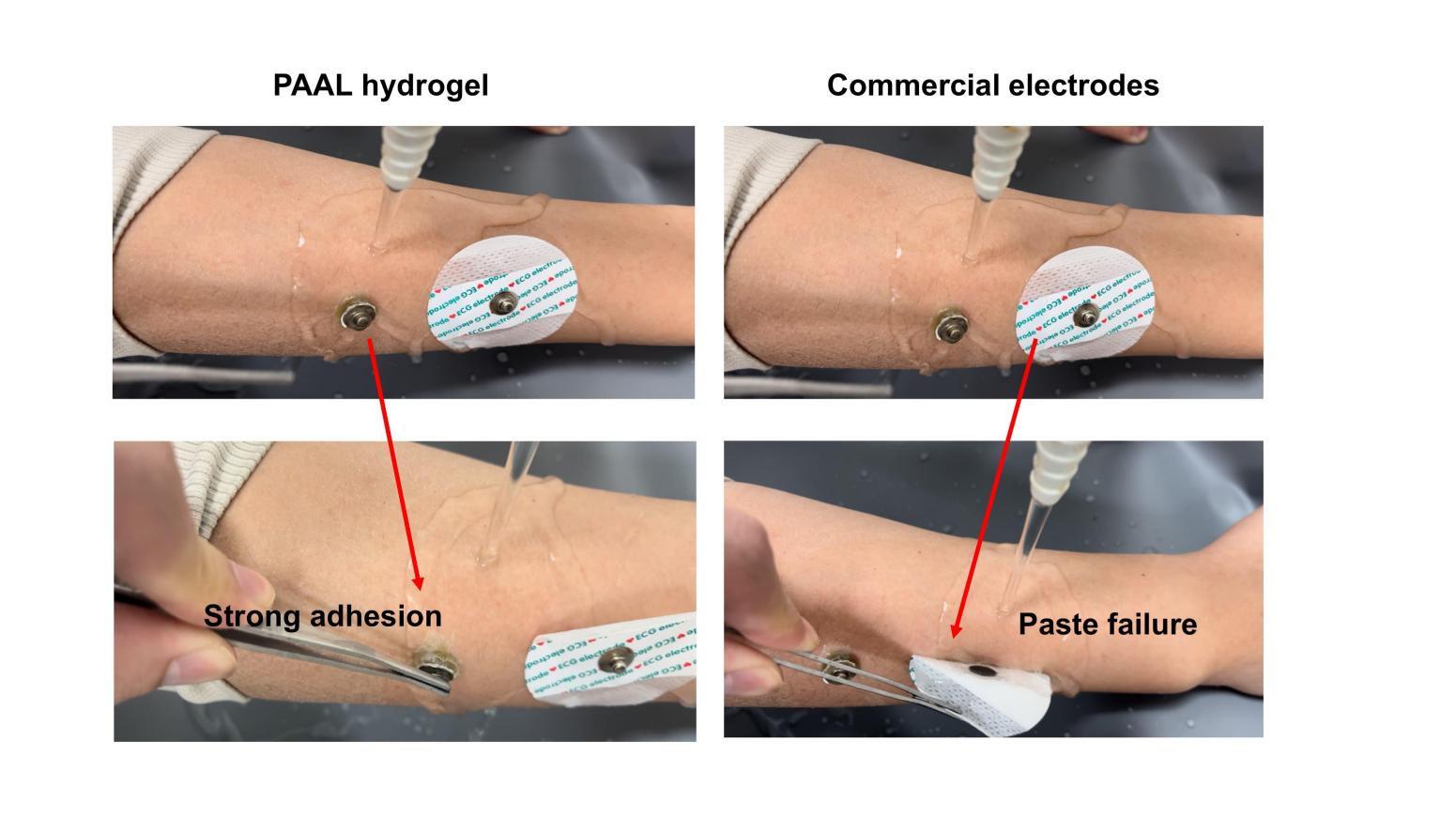
**

**Figure S16.** The commercial electrodes and PAAL hydrogel electrodes adhered to the surface of the arms were rinsed separately with a stream of water, and the commercial electrodes fell off quickly after wetting, while the PAAL hydrogel electrodes remain strongly adhered.


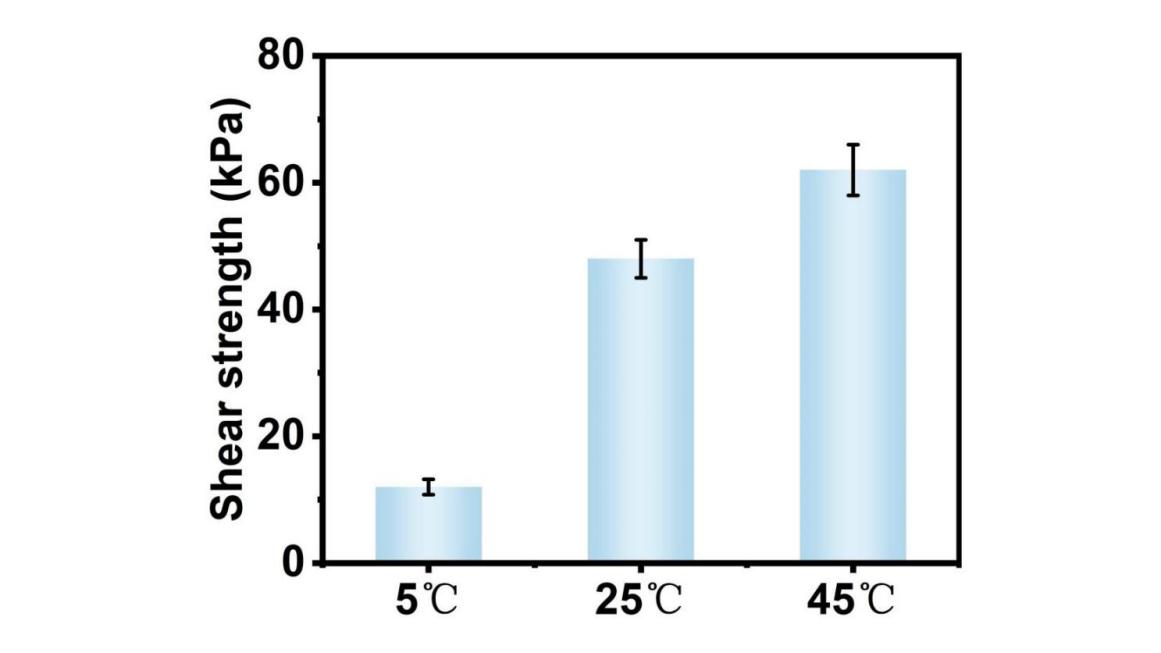


**Figure S17.** Shear adhesion strength of PAAL hydrogels at different temperatures.


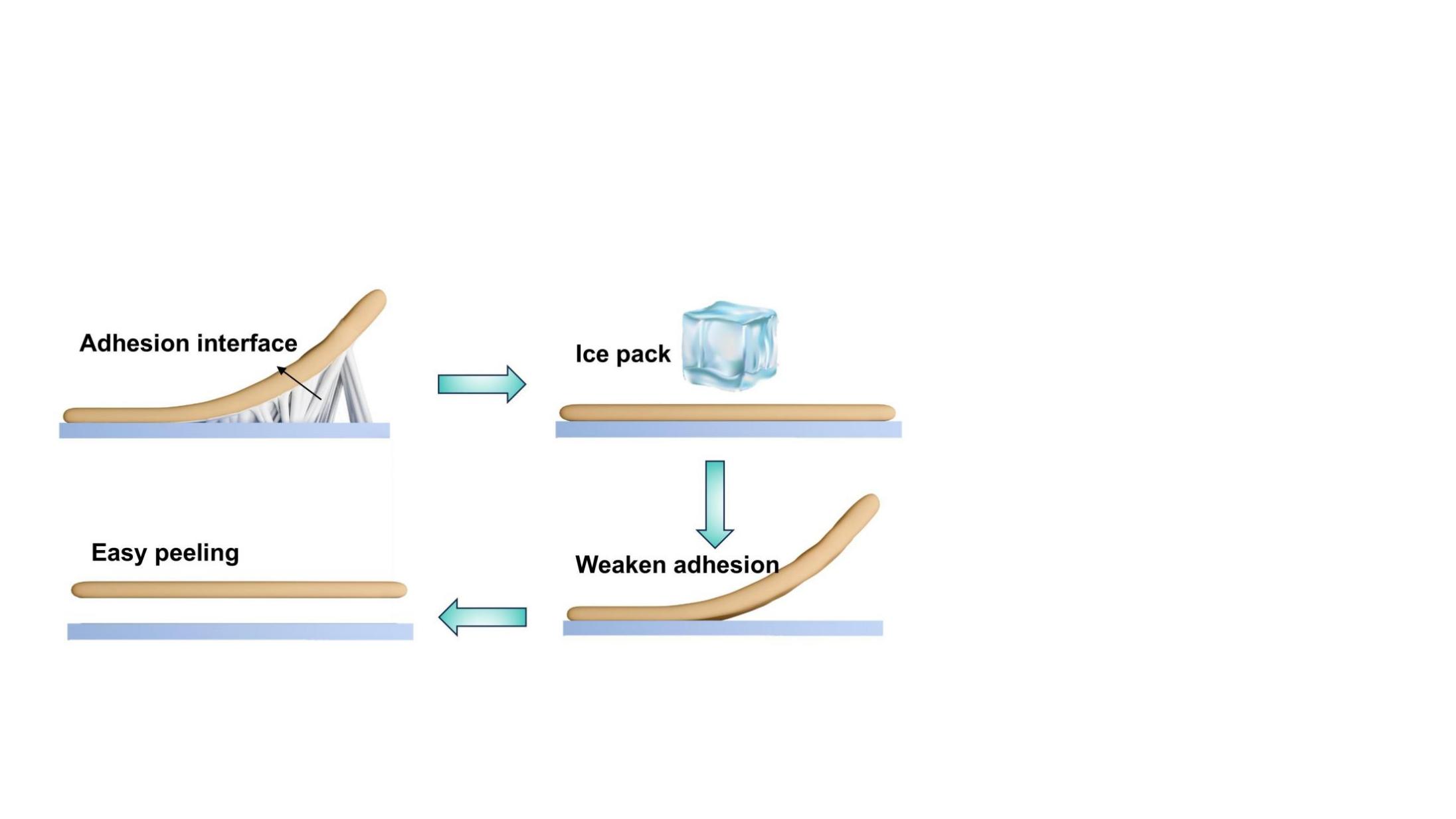


**Figure S18.** Schematic diagram of PAAL hydrogel subjected to cooling and de-adhesion transformation.


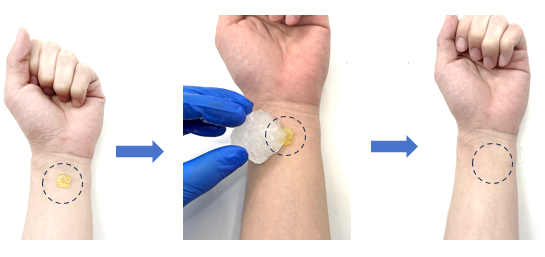


**Figure S19.** PAAL hydrogel adheres firmly to the skin with residue-free de-adhesion through ice.


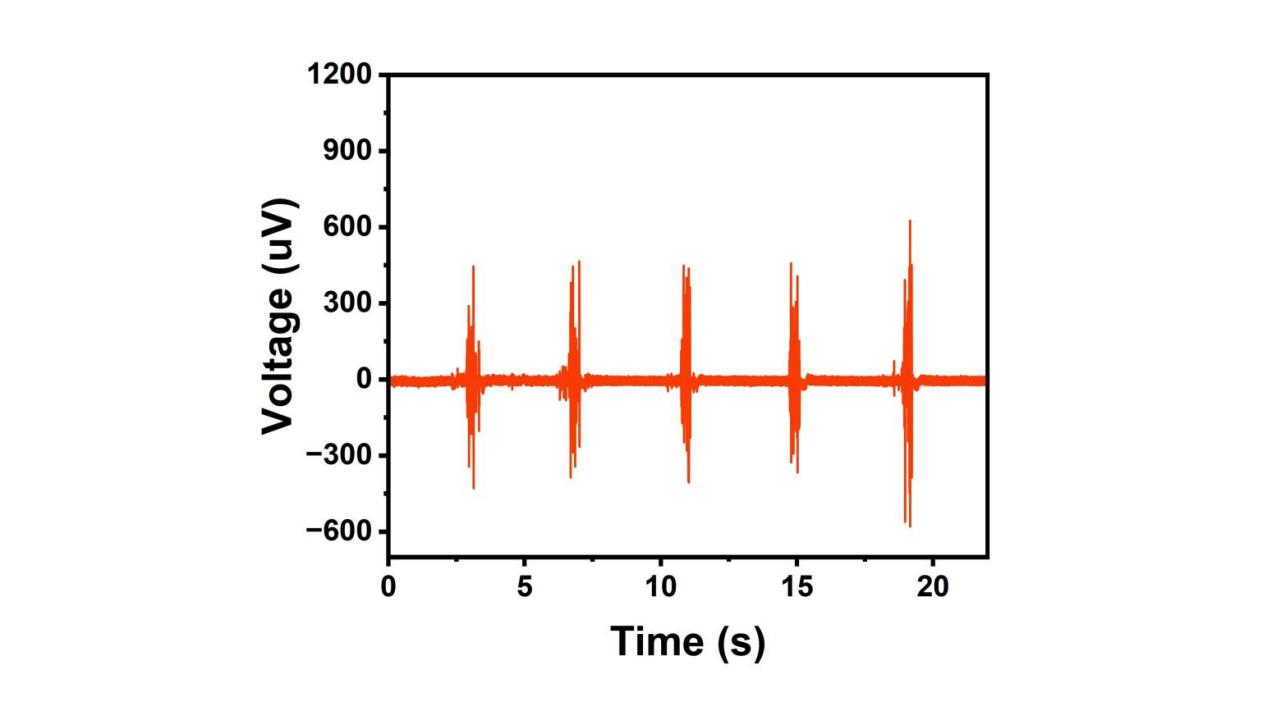


**Figure S20.** EMG signals acquired by PAAL hydrogel electrodes.


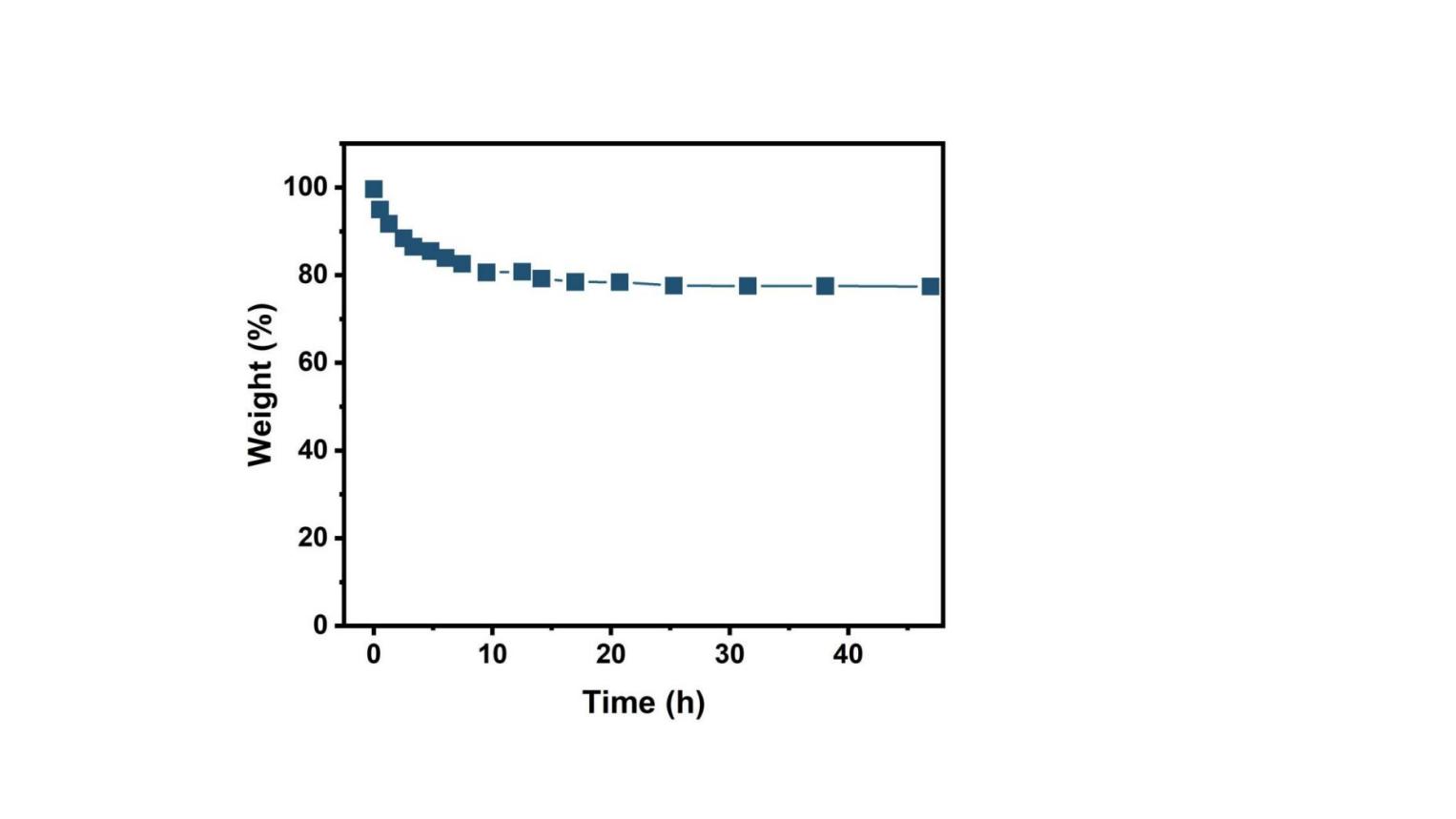


**Figure S21.** Rate of change in weight of PAAL hydrogel at 25°C room temperature.


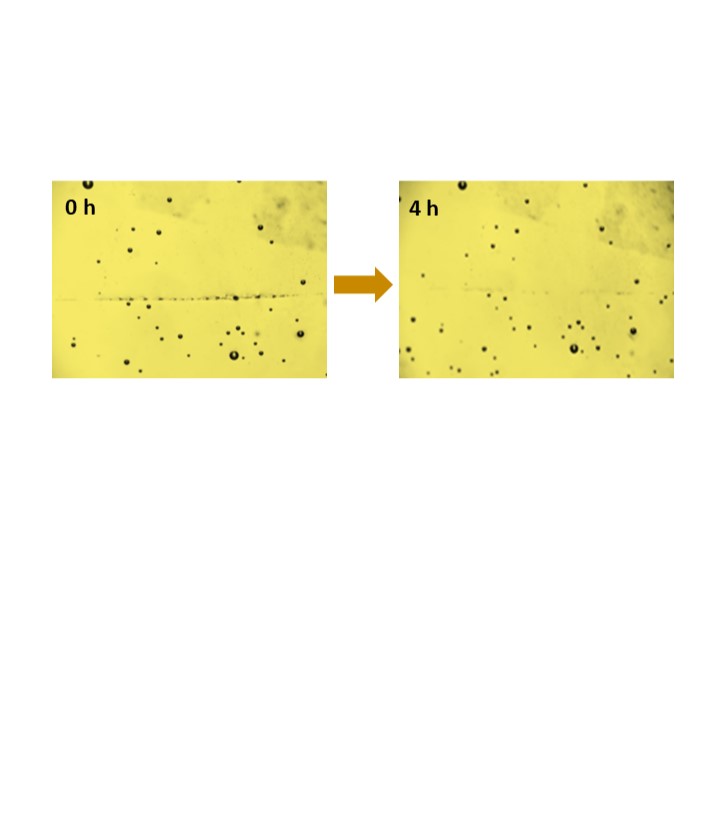


**Figure S22.** Self-healing scratch changes within 4 hours.


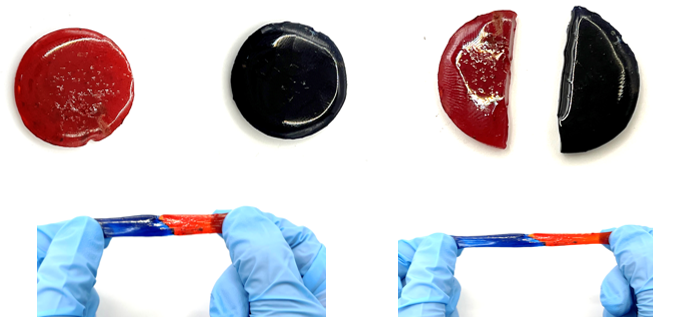


**Figure S23.** Self-healing process of stained PAAL hydrogel at room temperature.


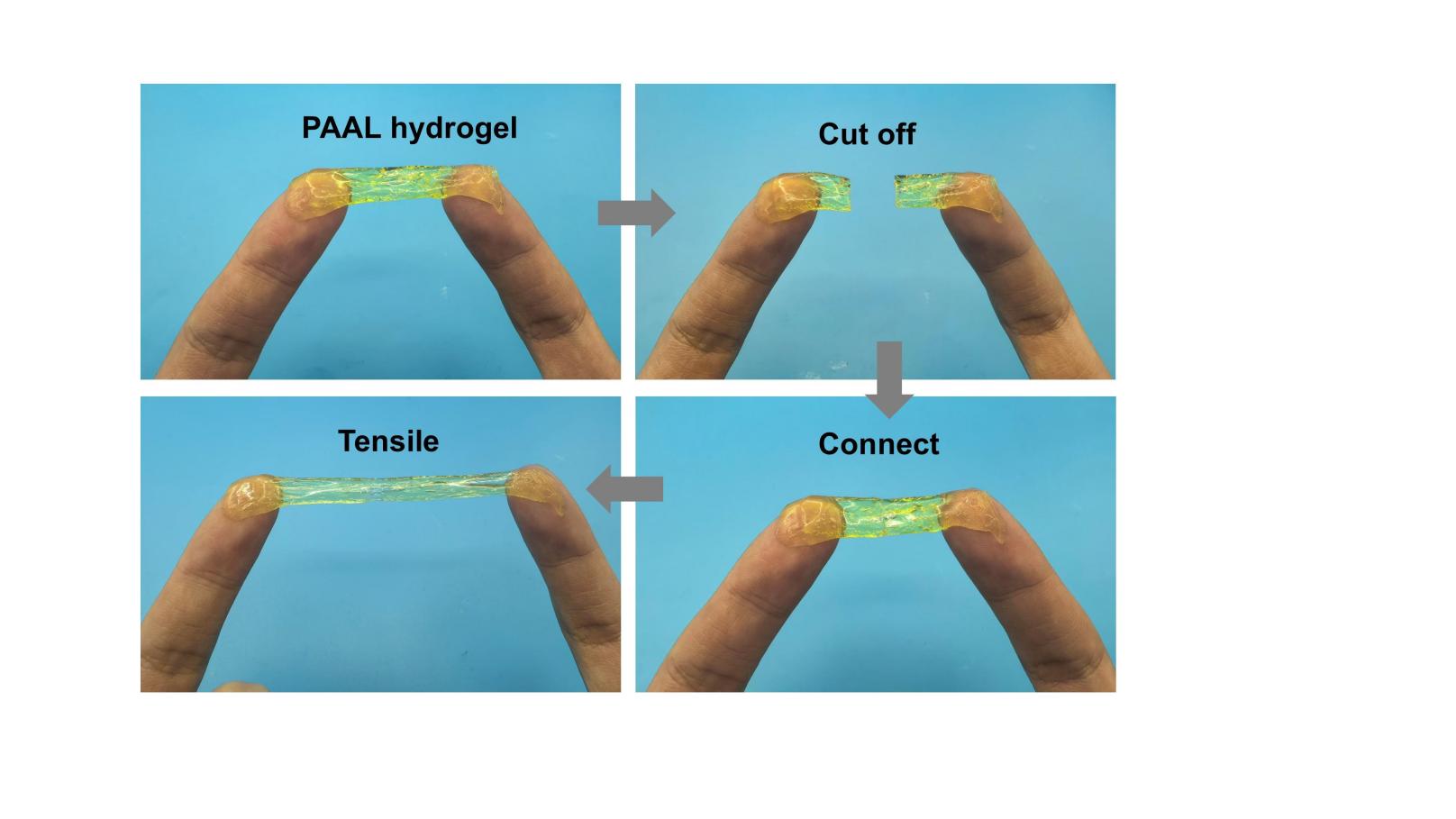


**Figure S24.** Demonstration of self-healing properties of PAAL hydrogel.


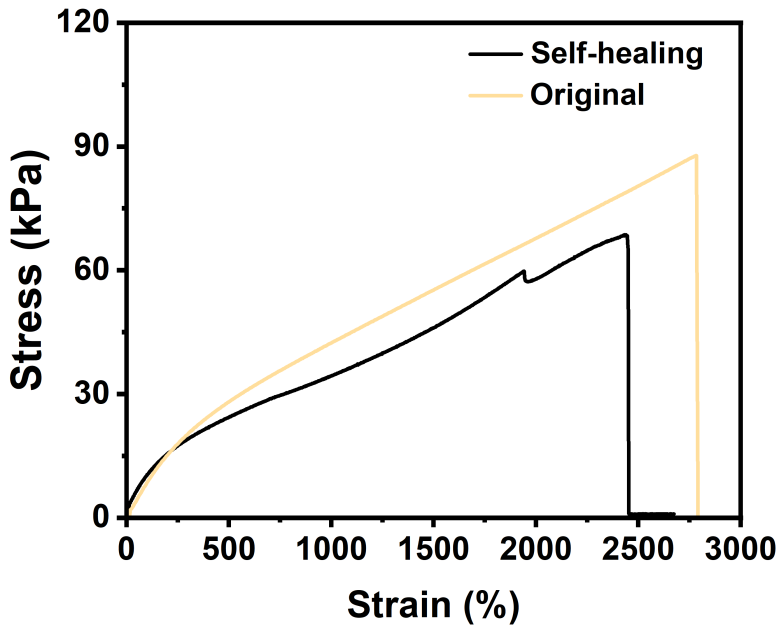


**Figure S25.** Stress-strain curves of the healed hydrogel versus the original hydrogel.


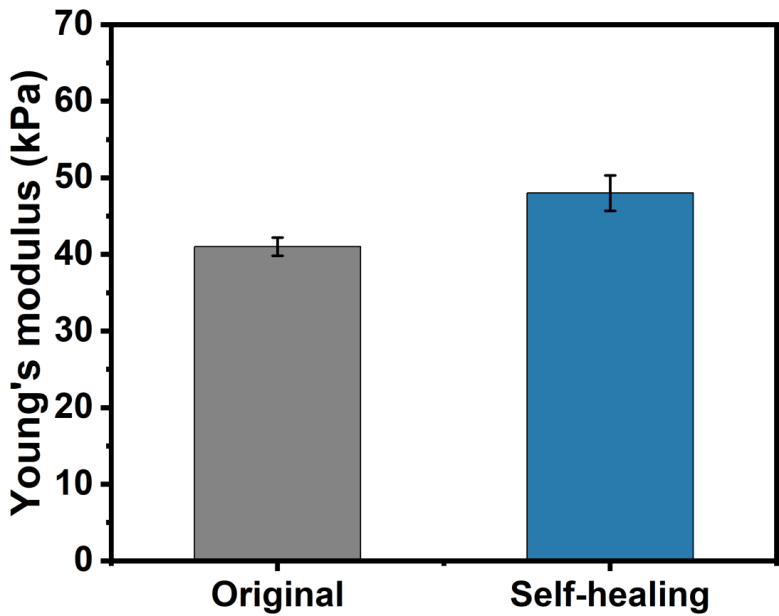


**Figure S26.** Young's modulus of the healed hydrogel versus the original hydrogel.


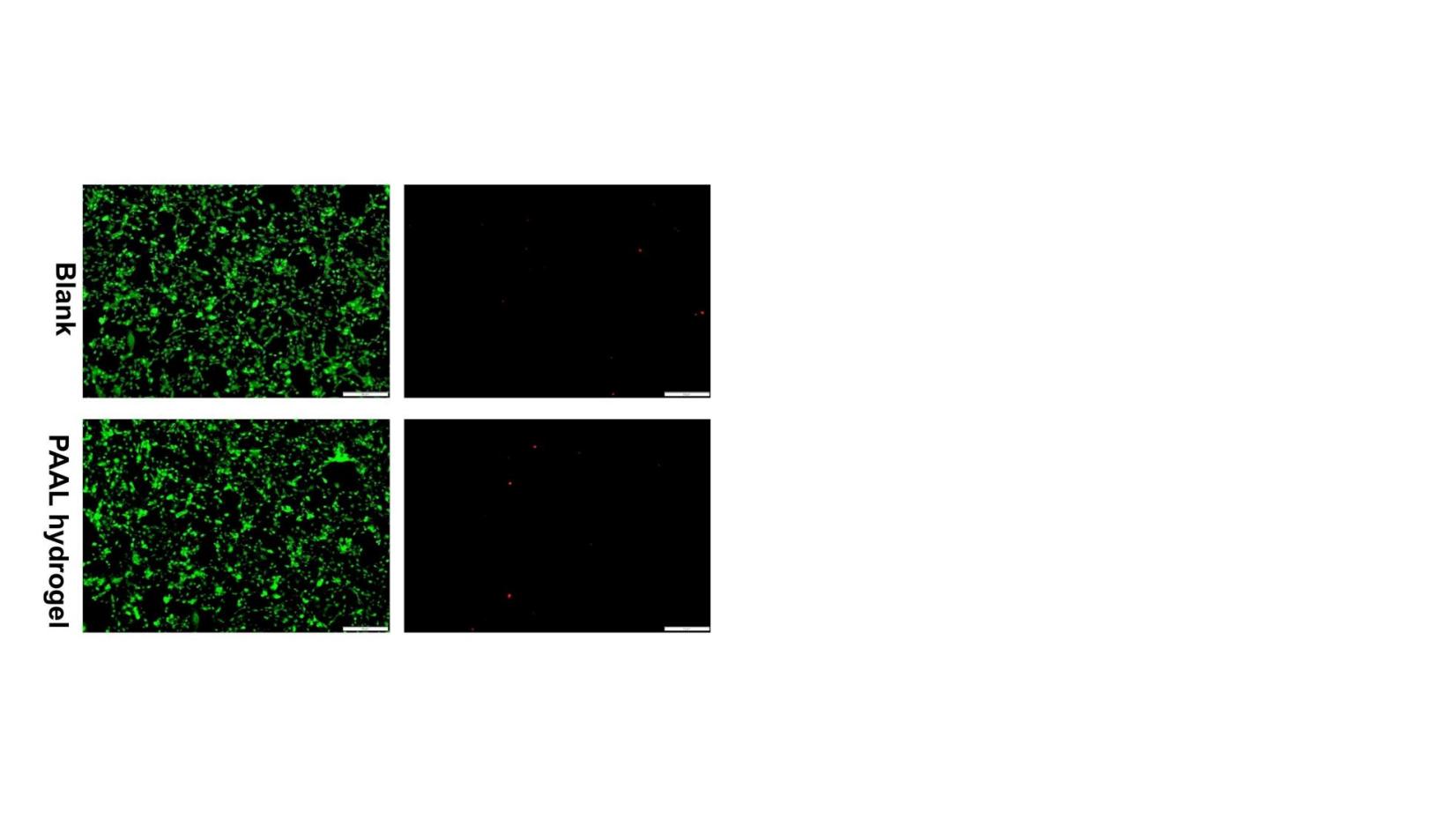


**Figure S27.** Fluorescence microscopy images of L929 live/dead cell double staining after co-culture with PAAL hydrogel (scale bar: 100 μm).


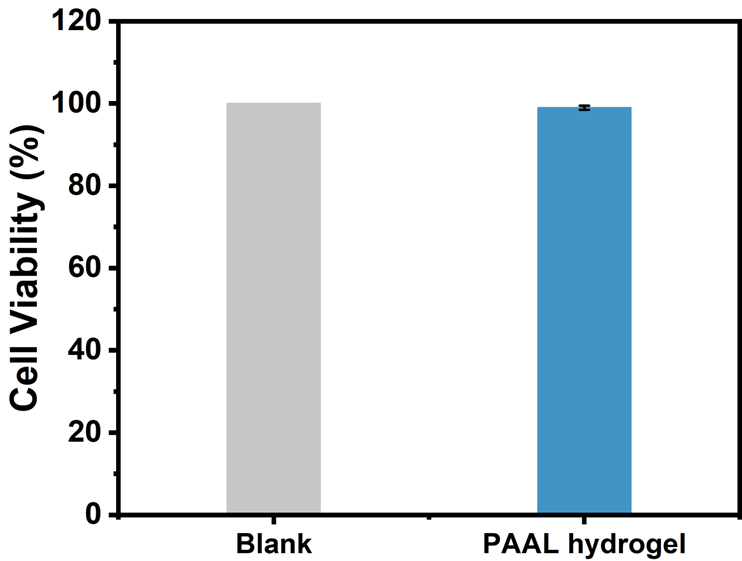


**Figure S28.** Survival of L929 cells after hydrogel treatment.


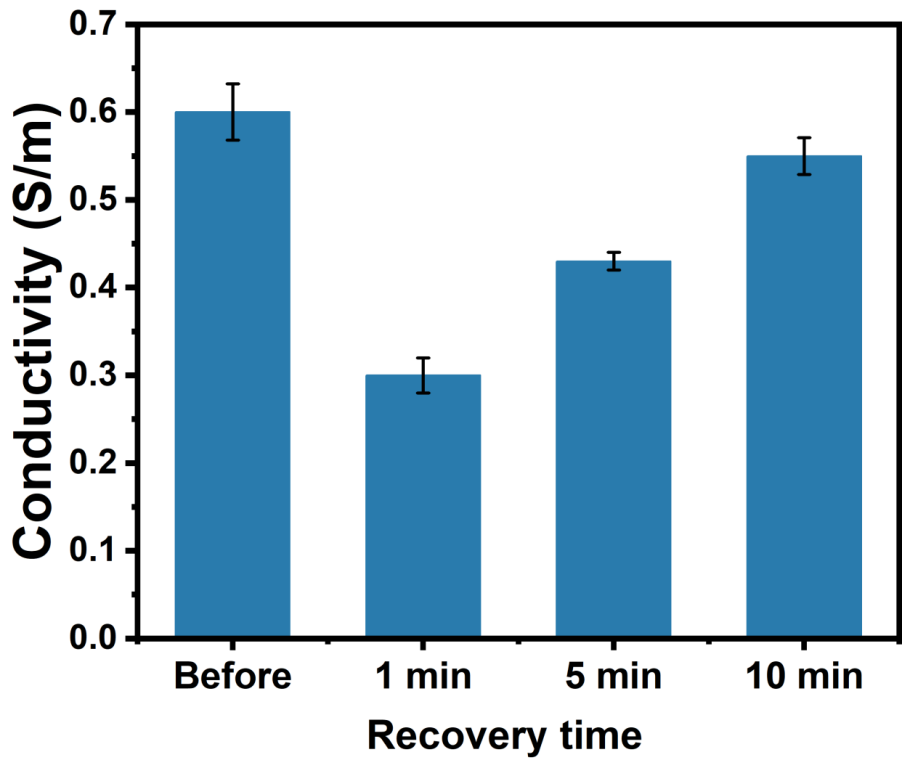


**Figure S29.** Electrical conductivity of PAAL hydrogels with different post-damage repair times.


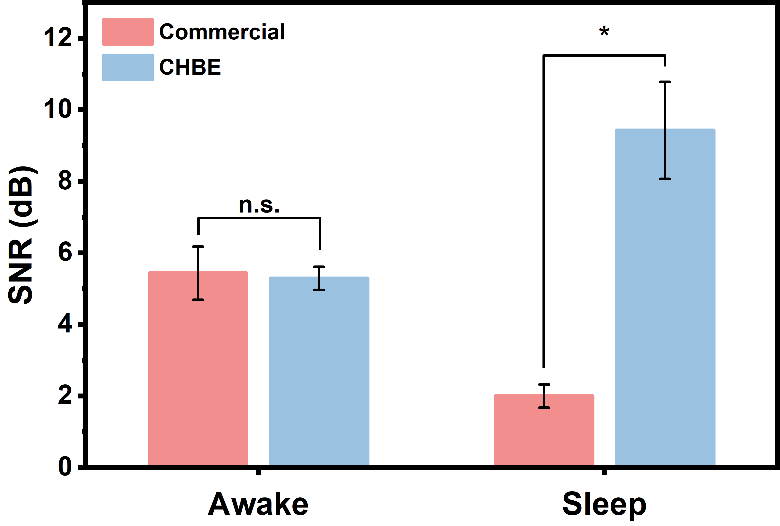


**Figure S30.** SNR of Delta and Theta waves in ECoG signals recorded from rats during awake and sleep states using CHBE and commercial stainless-steel electrodes, *p ≤ 0.05.


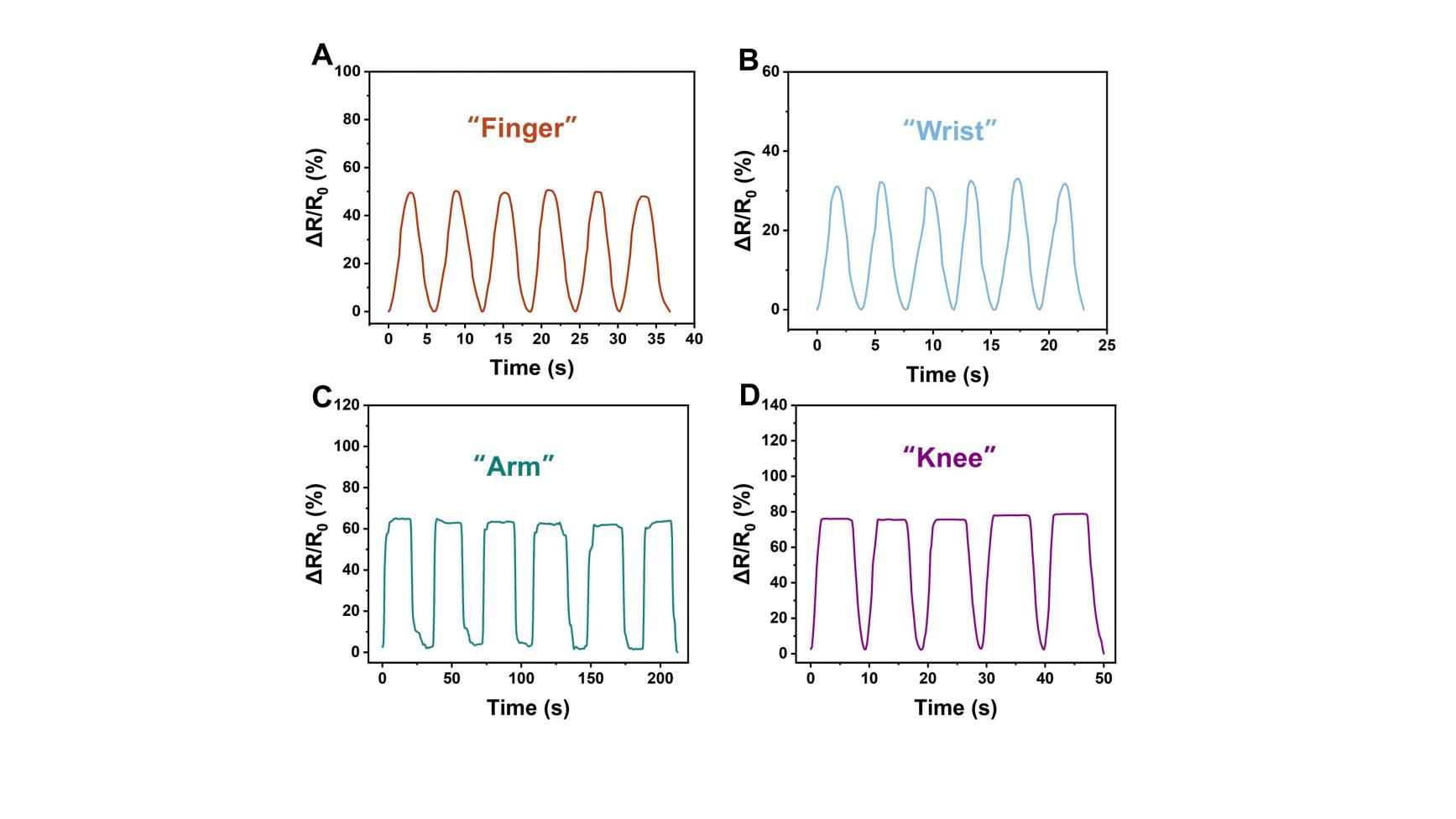


**Figure S31.** Sensing signals from different parts of the human body (finger, wrist, arm, and knee) area.


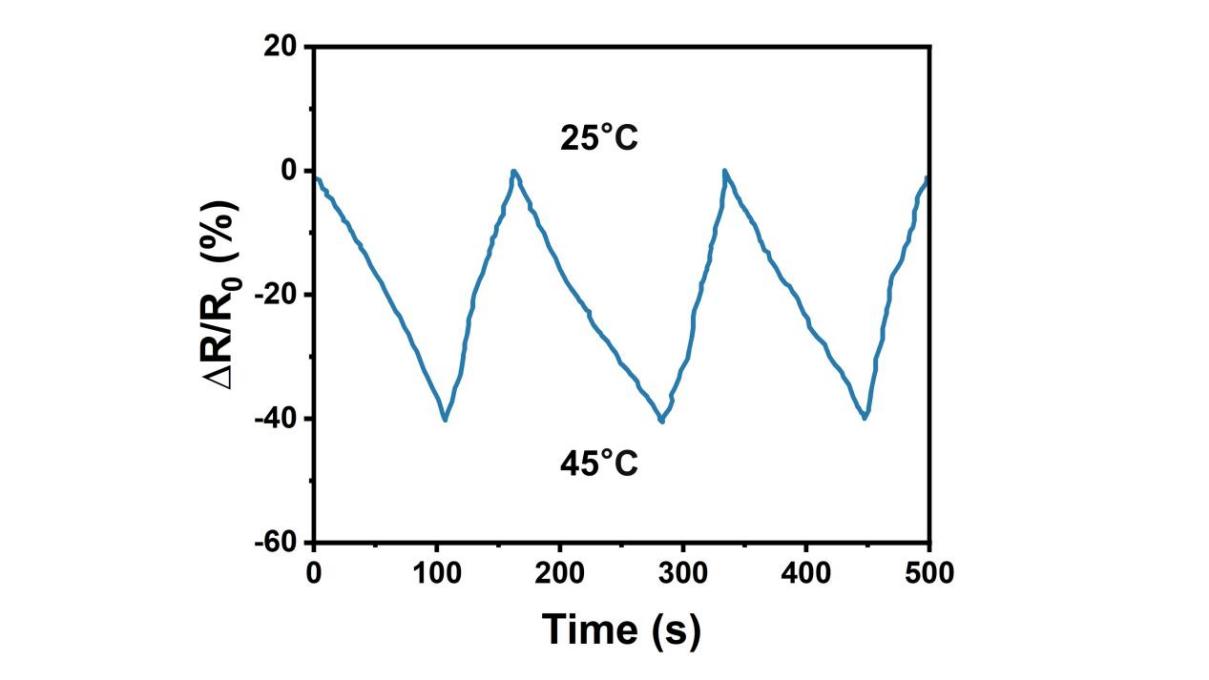


**Figure S32.** PAAL hydrogel as a temperature sensor.


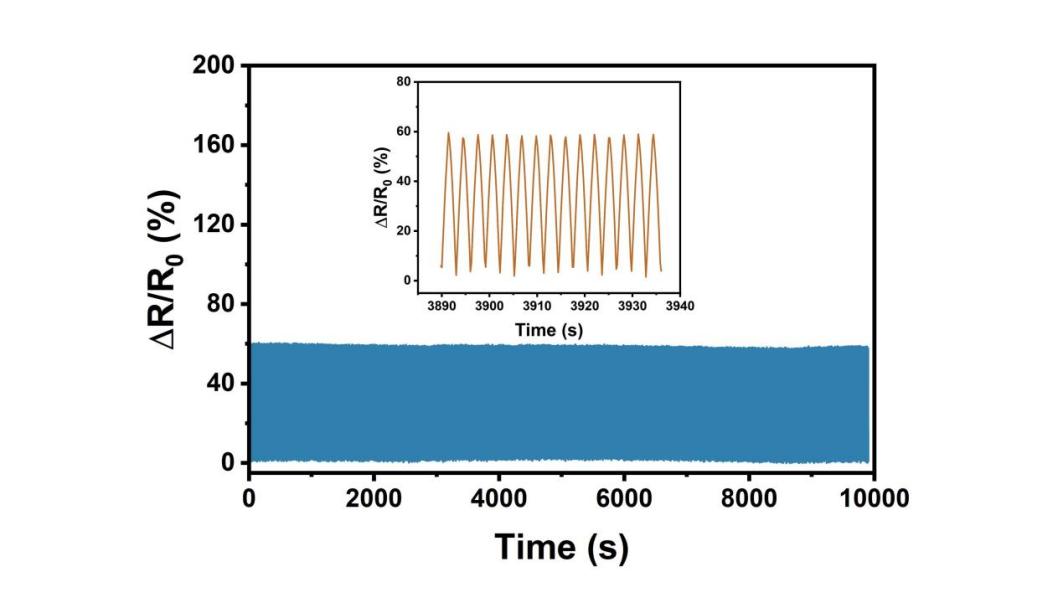


**Figure S33.** Resistance changes of repetitively stretched hydrogels at fixed strain.


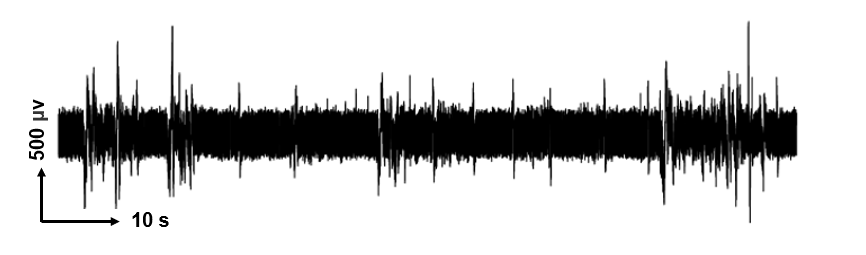


**Figure S34.** ECoG signals acquired by PAAL hydrogel electrodes.


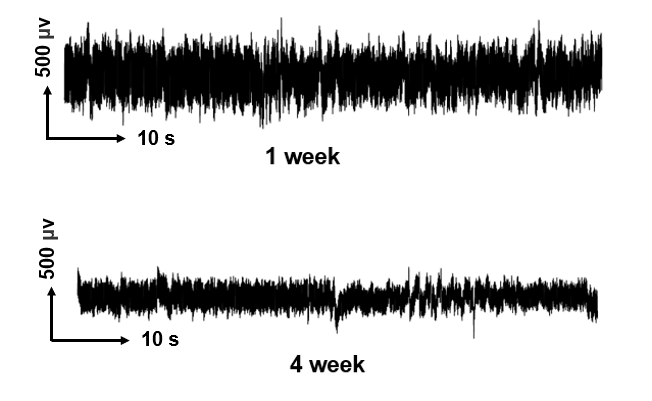


**Figure S35.** Recording of ECoG signals in the sleeping state by PAAL electrodes for four weeks.


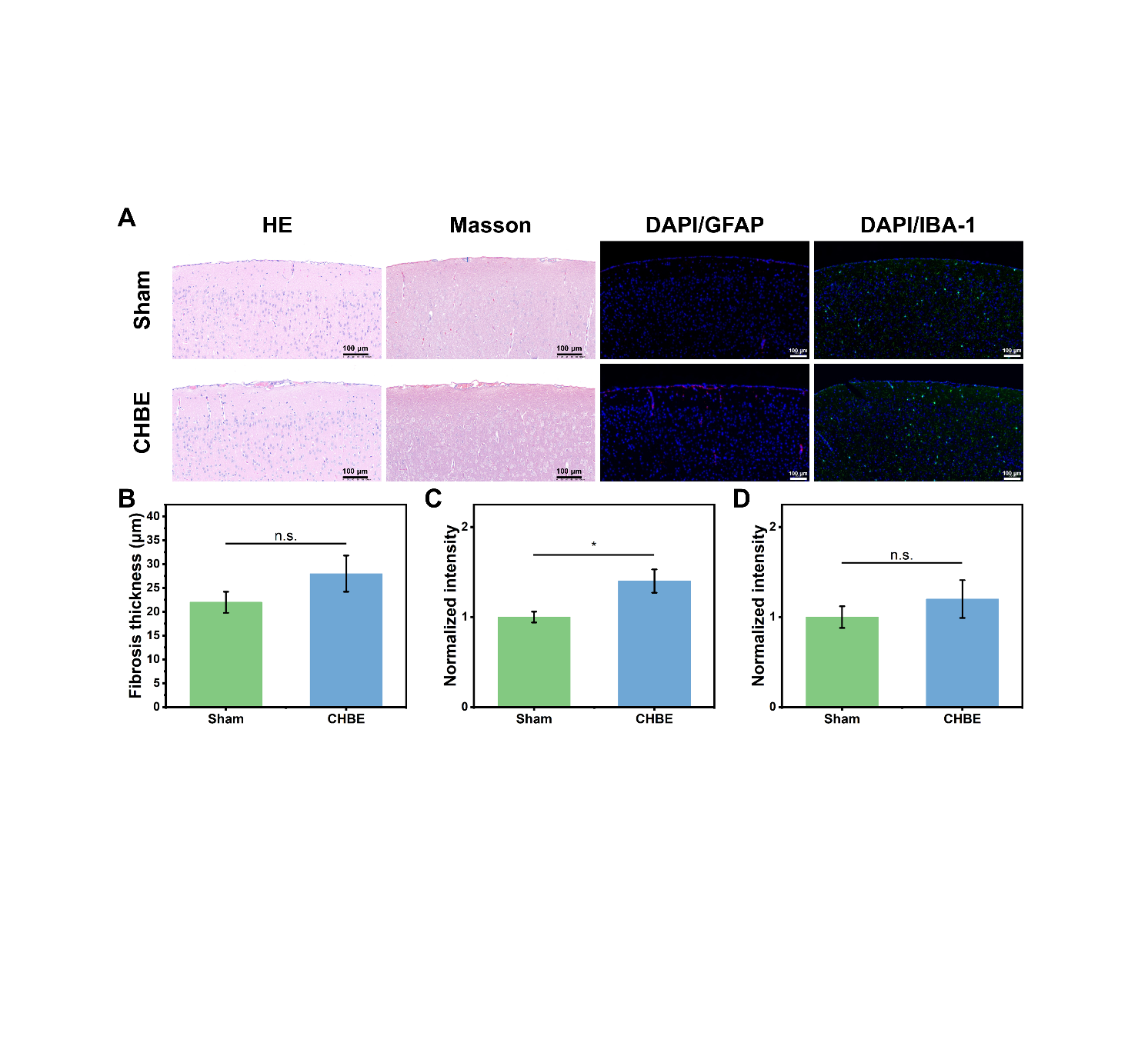


**Figure S36.** Biocompatibility assessment of CHBE after four weeks of ECoG signal acquisition. A) H&E staining, Masson’s staining, and immunofluorescence staining (DAPI/GFAP and DAPI/IBA-1) of brain tissue sections (n = 5) from the sham group and the CHBE-implanted group. The results show no significant differences in fibrous membrane thickness between the two groups, and only a slightly increased recruitment of glial cells in the CHBE group. B) Quantification of fibrotic capsule thickness in the sham and CHBE groups. C) Normalized GFAP fluorescence intensity relative to the sham group, *p ≤ 0.05. D) Normalized IBA-1 fluorescence intensity relative to the sham group.


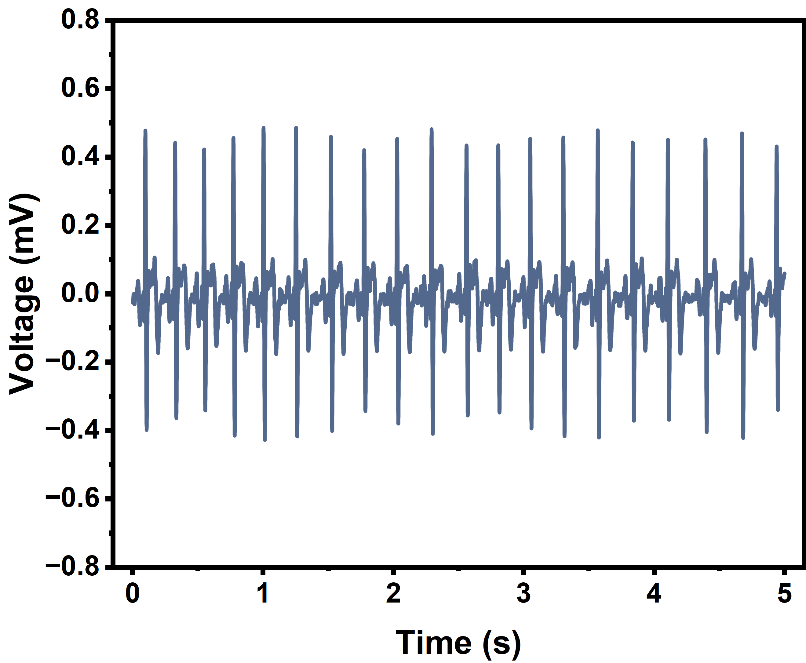


**Figure S37.** ECG signals obtained from rigid commercial electrodes.


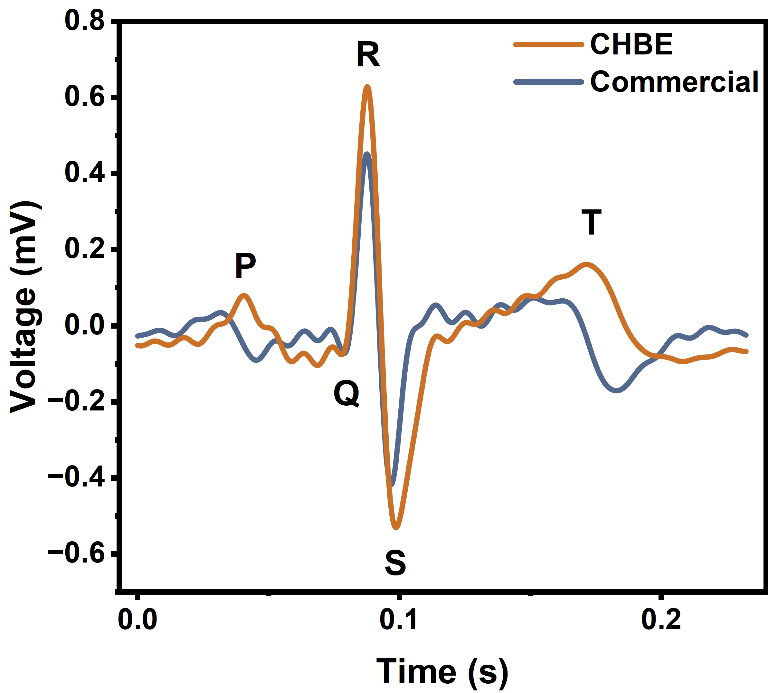


**Figure S38.** Comparison of ECG signals acquired from CHBE electrodes and commercial electrodes.


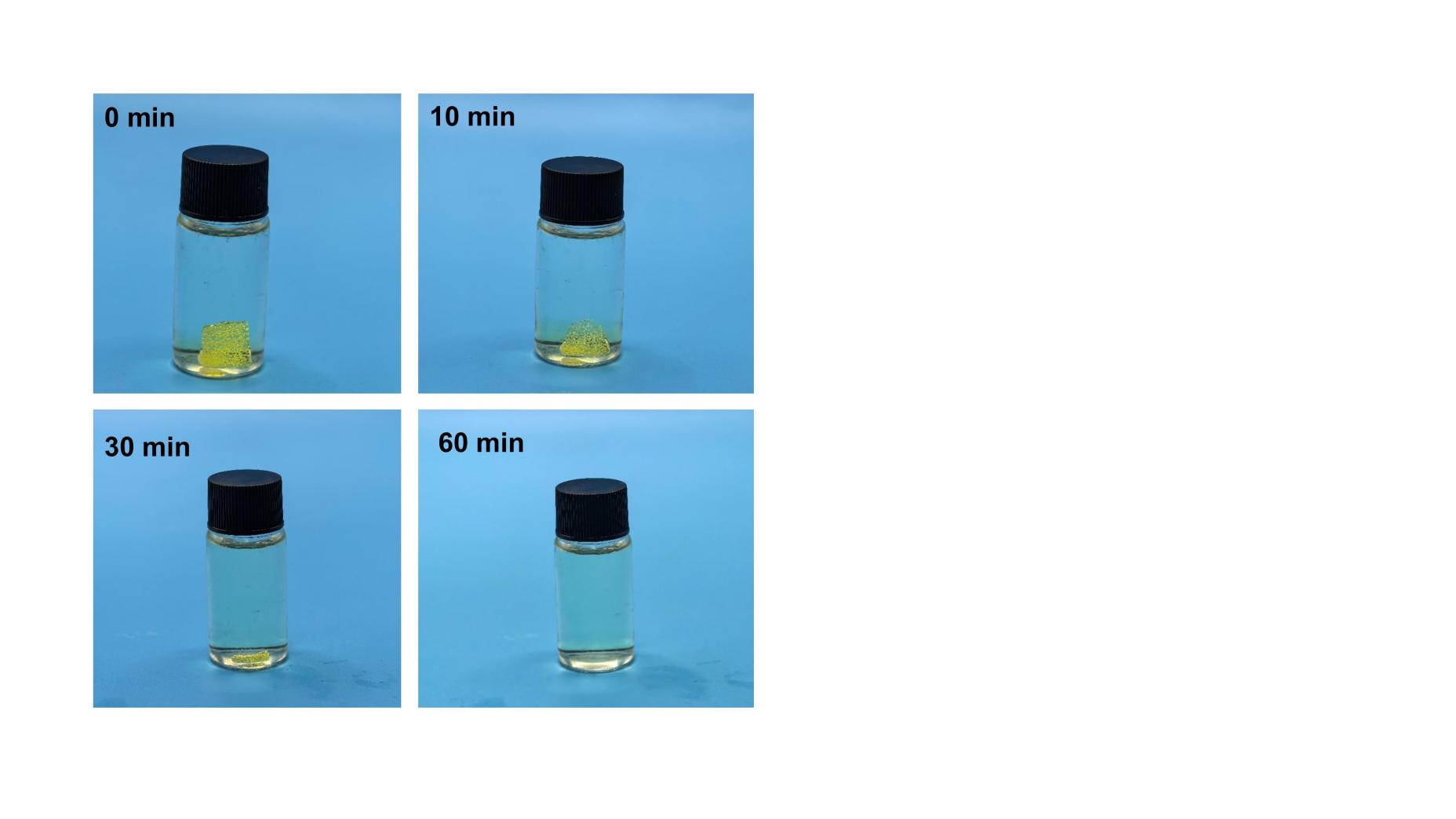


**Figure S39.** Degradation process of PAAL hydrogel in 0.5 M NaOH solution.

**Supplementary Table**

**Table S1**. Comparison with other reported hydrogel adhesives in various environments.

| **Types of materials** | **Processing mode** | **Adhesion environment** | **Under adhesion** | **Refs** |
| --- | --- | --- | --- | --- |
| P(AA-PLA-LA-NHS) | Hydrophilic/Hydrophobic segment migration | Water | 53.4 kPa | This work |
| PAA/Gelatin | Polymer template hydrophobicity | Water | 30.0 kPa | [1] |
| CMC-DA/TA | π-π stacking and hydrogen bond | Water | 45.9 kPa | [2] |
| P(AAc-co-AAm)/CTS/PEA | Hydrophobic association and electrostatic action | Water | 58.0 kPa | [3] |
| PVA/SA/TA/Ca^2+^ | Covalent bond | Air | 16.9 kPa | [4] |
| EPL/Tetra-PEG-SS | Hydrophilic/Hydrophobic association | Water, oil, and organic | 29.4 kPa | [5] |
| DA/PrGO | π-π stacking and metal coordination | Water | 4.6 kPa | [6] |
| P(AAc-co-AAm) | Structuralization | Water | 57.2 kPa | [7] |

**References**

[1] B. Yi, T. Li, B. Yang, S. Chen, J. Zhao, P. Zhao, K. Zhang, Y. Wang, Z. Wang, L. Bian, Surface hydrophobization of hydrogels via interface dynamics-induced network reconfiguration, Nat. Commun. 15 (2024) 239. https://doi.org/10.1038/s41467-023-44646-5.

[2] H. Xie, G. Shi, R. Wang, X. Jiang, Q. Chen, A. Yu, A. Lu, Bioinspired wet adhesive carboxymethyl cellulose-based hydrogel with rapid shape adaptability and antioxidant activity for diabetic wound repair, Carbohydr. Polym. 334 (2024) 122014. https://doi.org/10.1016/j.carbpol.2024.122014.

[3] G. Zhao, A. Zhang, X. Chen, G. Xiang, T. Jiang, X. Zhao, Barnacle inspired strategy combined with solvent exchange for enhancing wet adhesion of hydrogels to promote seawater-immersed wound healing, Bioact. Mater. 41 (2024) 46–60. https://doi.org/10.1016/j.bioactmat.2024.07.011.

[4] R. Song, X. Wang, M. Johnson, C. Milne, A. Lesniak-Podsiadlo, Y. Li, J. Lyu, Z. Li, C. Zhao, L. Yang, I. Lara-Sáez, S. A, W. Wang, Enhanced Strength for Double Network Hydrogel Adhesive Through Cohesion-Adhesion Balance, Adv. Funct. Mater. 34 (2024) 2313322. https://doi.org/10.1002/adfm.202313322.

[5] Y. Yang, G. He, Z. Pan, K. Zhang, Y. Xian, Z. Zhu, Y. Hong, C. Zhang, D. Wu, An Injectable Hydrogel with Ultrahigh Burst Pressure and Innate Antibacterial Activity for Emergency Hemostasis and Wound Repair, Adv. Mater. 36 (2024) 2404811. https://doi.org/10.1002/adma.202404811.

[6] H. Wei, H. Jing, C. Cheng, Y. Liu, J. Hao, A Biomimetic One-Stone-Two-Birds Hydrogel with Electroconductive, Photothermally Antibacterial and Bioadhesive Properties for Skin Tissue Regeneration and Mechanosensation Restoration, Adv. Funct. Mater. 35 (2025) 2417280. https://doi.org/10.1002/adfm.202417280.

[7] Y. Wang, D. Liu, C. Wang, J. Wu, X. Xu, X. Yang, C. Sun, P. Jiang, X. Wang, 3D printing of octopi-inspired hydrogel suckers with underwater adaptation for reversible adhesion, Chem. Eng. J. 457 (2023) 141268. https://doi.org/10.1016/j.cej.2022.141268.
